# On using non-Kekulé triangular graphene quantum dots for scavenging hazardous sulfur hexafluoride components

**DOI:** 10.1016/j.heliyon.2023.e15388

**Published:** 2023-04-07

**Authors:** Vaishali Roondhe, Basant Roondhe, Sumit Saxena, Rajeev Ahuja, Alok Shukla

**Affiliations:** a Department of Physics, Indian Institute of Technology Bombay, Mumbai 400076, Maharashtra, India; b Department of Metallurgical Engineering and Materials Science, Indian Institute of Technology Bombay, Mumbai 400076, Maharashtra, India; c Materials Theory Division, Department of Physics and Astronomy, Uppsala University, Box 516, Uppsala 75120, Sweden; d Department of Physics, Indian Institute of Technology Ropar, 140001, Punjab, India

**Keywords:** Graphene quantum dot, Sulfur hexafluoride, Density functional theory, Chemical functionalization, Electronic properties

## Abstract

The goal of present study is to explore how the size and functionalization of graphene quantum dots (GQDs) affect their sensing capabilities. Specifically, we investigated the adsorption of SO_2_, SOF_2_, SO_2_F_2_, and SF_6_ on GQDs that were functionalized with –CH_3_, –COCH_3_, and –NH_2_. We used density functional theory to analyse the electronic properties of these functionalized GQDs and found that the functionalization significantly altered their electronic properties. For example, the B3LYP H-L gap of pristine triangulene was 3.9eV, while the H-L gap of functionalized triangulene ranged from 2.8 eV to 3.6 eV (using the B3LYP functional). Our results indicate that –NH_2_ functionalized phenalenyl and triangulene provide strong interaction with SO_2_, with adsorption energies of −0.429 eV and −0.427 eV, respectively. These adsorption properties exhibit physisorption, leading to high gas sensitivity and superior recovery time. The findings of this study provide new insights into the potential use of GQDs for detecting the decomposed constituents of sulfur hexafluoride, which can be beneficial for assessing the operation status of SF_6_ insulated devices. Overall, our calculations suggest that functionalized GQDs can be employed in gas insulated systems for partial discharge detection.

## 1 Introduction

Sulfur hexafluoride (SF_6_) is an odourless, nontoxic, non-flammable, colourless and inert gas that possesses excellent insulation and strong chemical stability [1,2]. Its dielectric strength and chemical inertness make it a crucial medium for electrical insulators and in gas-insulated switchgear (GIS) for power systems as an arc-quenching medium [3,4]. However, in certain cases, partial discharge can occur when the electric field is intensified over time, leading to the decomposition of SF_6_. The resulting products, such as SO_2_, H_2_S, SO_2_F_2_, CF_4_, and SOF_2_, are responsible for equipment corrosion and can potentially lead to system failure [[5], [6], [7], [8], [9]]. Current methods used to detect these decomposed products of SF_6_ include ultrasonic, ultrahigh frequency, gas sensing and optical measurement methods [[10], [11], [12]]. Among these, the gas sensing method is highly recommended due to its high sensitivity in less volume and the most important thing, it is cost effective [[13], [14], [15], [16]]. However, gases such as SO_2_, SOF_2_, and SO_2_F_2_, which have extremely low concentrations on the order of parts per million (PPM), are challenging to detect with conventional sensors [17].

Nanomaterials based on graphene have received significant attention in the scientific community following the successful fabrication of graphene [18]. For both fundamental science and practical applications, graphene oxide (GO), reduced graphene oxide (rGO), and graphene quantum dots (GQD) have been extensively studied [[19], [20], [21], [22]]. Within the graphene family, GQD possess superior properties such as chemical inertness, low cytotoxicity, size and shape dependent photoluminescence etc. [[23], [24], [25]], make them useful for various applications in electronics (super capacitor, flash memory etc.), optical (photodetector, phototransistor etc.), medical (drug delivery, cancer phototherapy etc.) and energy [[26], [27], [28], [29], [30], [31], [32]]. Due to large surface-to-volume ratio, GQD also have superior sensing properties [24,33] making them useful in electrical and optical gas sensing devices [33,34]. A group of GQD have closed-shell electronic structures, while some, due to their unique topology, may have open-shell structure with high-spin ground states [35,36]. Open-shell GQD with π-electrons near the Fermi level have a delocalized radical character, which is useful for spintronics [[37], [38], [39]] and energy-related applications [40]. Closed-shell graphene fragments have closed-shell electronic configuration (no unpaired electrons) with π**-**electrons in bonding orbitals. However, open-shell graphene fragments have unpaired electrons or partially unpaired electrons, which results in non-bonding single occupied molecular orbitals (SOMO) in molecular orbital theory. For example, graphene fragments with two unpaired electrons are also known as biradical hydrocarbons, in which the ground state has two nonbonding molecular orbitals filled by two unpaired electrons [41]. Open-shell graphene fragments are unique in nature, possessing intriguing optical, electronic, magnetic properties and crystalline structures compared to their closed-shell counterparts. As a result, open-shell graphene fragments have numerous potential applications in photovoltaic devices [42], spintronic devices [43,44] etc. As stated by Lambert, “The future of these biradical PAHs clearly lies in materials science” [45]. Although useful in their pristine form, oxygen (−OH, –O−, –OCH_3_, –COOH, etc.), amine (−NH_2_) and methyl (−CH_3_) functionalized GQDs generally have improved solubility and superior quantum yield (QY) due to changes in their electronic structure caused by functionalization [[46], [47], [48]].

The study of sensing SF_6_ decomposition products on GQDs is motivated by the need to monitor and detect the breakdown of SF_6_, a commonly used gas in electrical equipment such as switchgear and circuit breakers. SF_6_ is a powerful insulator and arc quencher, but it is also a potent greenhouse gas with a global warming potential of 23,500 times that of CO_2_. SF_6_ decomposition products can indicate the presence of partial discharges, corona discharges, or other electrical faults in the equipment, which can lead to equipment failure if left unaddressed. Sensing SF_6_ decomposition products on GQDs can provide a sensitive and selective method for detecting these breakdowns, and thus, enabling early maintenance and preventing equipment failure. In this work, we computationally study the sensing properties of three different GQDs (phenalenyl, triangulene, and extended triangulene) for gases SO_2_, SOF_2_, SO_2_F_2_ and SF_6_ using a DFT methodology.

## 2 Computational methodology

As mentioned earlier, we employed the Gaussian16 suit of programs [49] to perform all the DFT calculations presented in this work. In the first step, we optimized the geometries of –CH_3_, –COCH_3_ and –NH_2_ functionalized phenalenyl, triangulene and extended triangulene. In the simulation process, the charge is neutral for all the considered structures along with the molecules adsorbated. The multiplicity of doublet, triplet and quartet is utilized for phenalenyl, triangulene and extended-triangulene, respectively, due to presence of one, two and three unpaired electrons. To address the significant role played by van der Waals (vdW) interactions in systems involving adsorption, we utilized the ωB97XD functional. This functional incorporates London dispersion corrections that account for the long-range interactions that arise during the adsorption process. The ωB97XD functional includes Grimme's van der Waals (vdW) correction term of –C_6_/R^6^, also known as the GD2 dispersion model. The mathematical expression for the dispersion correction term is as follows:

(1) $$E_{disp}^{D2}=-S_{6}\sum_{i>j}^{N_{atoms}}{\frac{C_{6}^{ij}}{{\left(R_{ij}\right)}^{6}}f}_{damp}\left(R_{ij}\right)$$

Here, $f_{damp}\left(R_{ij}\right)=\frac{1}{1+{a\left(R_{ij}/R_{r}\right)}^{-12}}$ represents the damping function. $R_{r}$ is the sum of vdW radii of the atomic pair *ij*, and the parameter, a, governs the power of dispersion corrections. The number of atoms in the system is denoted by $N_{atoms}$ in Eq. (1). Additionally, $C_{6}^{ij}$ represents the dispersion coefficient for the atom pair ij, while $R_{ij}$ denotes the interatomic distance between them. The value of the fitting parameter S_6_, used in the damping function to account for the correlation of this additive dispersion term, was included in the ωB97XD functional, and set to 1.0 [50].

In addition, we utilized the hybrid functional B3LYP [51,52], which is a combination of the Becke exchange functional (Becke three parameter) with the Hartree-Fock exchange term. The B3LYP hybrid functional incorporates a non-local correlation functional from LYP (Lee, Yang and Parr) and a local correlation functional of the form VWN (Vosko, Wilk, and Nusair). We employed a 6-31G (D) split-valence basis set, which consists of six Gaussian functions for describing inner-shell orbitals and a split-valence set of four Gaussians for the valence orbitals, with subsets of 3 and 1. The gases to be adsorbed were initially placed parallel to the surfaces of the functionalized GQDs, and the whole systems (GQD + adsorbed gas) were then permitted to relax until the gradient forces achieved the predetermined threshold of 0.00045 Hartree. The optimized structure and the molecular orbitals were visualized with GaussView (version 6). The adsorption energy (E_ad_) of SO_2_, SOF_2_, SO_2_F_2_ and SF_6_ molecules on the three functionalized GQDs was computed using the equation [53,54]:

(2) $$E_{ad}=E_{g+GQDs}-\left(E_{g}+E_{GQDs}\right)$$

where $E_{g+GQDs}$ is the optimized total energy for the gas molecule g = SO_2_, SOF_2_, SO_2_F_2_ and SF_6_ adsorbed over GQDs = phenalenyl, triangulene and extended triangulene, $E_{g}$ is the optimized total energy of individual gas molecule g and $E_{GQDs}$ is the optimized energy of pristine GQDs (phenalenyl, and triangulene and extended triangulene). Using the definition, negative value of $E_{ad}$ value shows a stable adsorption complex on the GQD. To further check the effect of higher basis set, we have evaluated the $E_{ad}$ using def2-TZVPP triple zeta basis set and the discussion is presented in supplementary material. The formation energy *E*_*f*_ are calculated using equation:

(3) $$E_{f}=\frac{1}{N}[E_{functionalized\;GQDs}-nE_{c}-nE_{H}-nE_{O/N}]$$

where $E_{functionalized\;GQDs}$ is the total energy of the functionalized phenalenyl, triangulene and extended triangulene system, $E_{c},E_{H}$ and $E_{O/N}$ is the total energy of individual carbon, hydrogen, oxygen or nitrogen. N represents total number of atoms in the system while, n represents individual number of atom respectively. We observed negative formation energy for all systems, conforming thermodynamical stability and also suggest finite possibility of these materials to be synthesize experimentally. The results obtained using ωB97XD functional are presented in the main text, while those corresponding to B3LYP are presented in the supplementary material.

## 3 Results and discussion

In this study, we focus on three triangular GQDs with unique topology (i) phenalenyl, which has three fused benzene rings and a total spin quantum number of 1/2, (ii) triangulene which has six fused benzene rings and a total spin quantum number of, and (iii) extended triangulene, which has ten fused benzene rings and a total spin quantum number of 3/2. These GQDs have unusual topologies that prevent the formation of an aromatic structure without the presence of unpaired electrons, resulting in high-spin ground states. Additionally, to investigate the effect of different functional groups on the sensing of gases SO_2_, SOF_2_, SO_2_F_2_ and SF_6_ gases, we functionalize phenalenyl, triangulene and extended triangulene with three different groups: methyl (CH_3_), ketone (COCH_3_) and amino (NH_2_). These functional groups are chosen based on previous experimental and theoretical studies [23,48,55]. For simplicity, phenalenyl with functional group is denoted as Phe + CH_3_, Phe + COCH_3_ and Phe + NH_2,_ triangulene with functional group is denoted as Tri + CH_3_, Tri + COCH_3_ and Tri + NH_2_, and the extended triangulene as Ex-Tri + CH_3_, Ex-Tri + COCH_3_ and Ex-Tri + NH_2_. Our results were obtained using the ωB97XD functional, which includes long-range corrections for a more accurate description of adsorption properties. The results of the B3LYP functional can be found in the supplementary material.

### 3.1 Structural properties

**Fig. 1:**
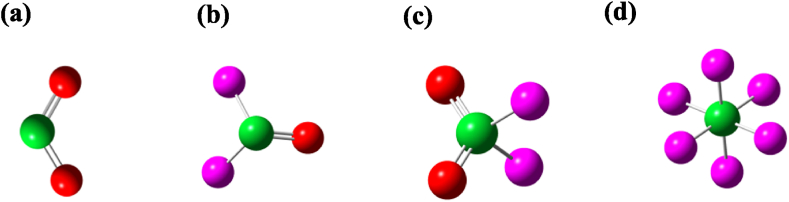
Optimized structures of SO_2_, SOF_2_, SO_2_F_2_, and SF_6_ molecules. The green, red and purple balls represent sulfur, oxygen and fluorine atoms respectively. (For interpretation of the references to colour in this figure legend, the reader is referred to the Web version of this article.)

Before examining the adsorption process, the structures of the four gas molecules and the three GQDs functionalized with various groups were optimized individually to obtain their most stable configurations. First, we will discuss the optimized structures of the individual gas molecules SO_2_, SOF_2_, SO_2_F_2_ and SF_6_ (see Fig. 1(a–d)). In the case of the SO_2_ molecule, the bond length of S–O bond is 1.469 Å with bond angle of 119.127°. In SOF_2_, sulfur is the centre atom bonding with both fluorine atoms and oxygen atom, comprising sp^2^ hybridization. The S–O and S–F bond lengths are 1.445 Å and 1.627 Å respectively. The F–S–O bond angle is 107.07°, while F–S–F bond angle is 92.652°. In the case of SO_2_F_2,_ sulfur is the centre atom bonding with both fluorine atoms and oxygen atoms leading to sp^3^ hybridization. The S–O and S–F bond lengths are 1.434 Å and 1.586 Å, respectively. In the case of SF_6_, sulfur is the central atom bonding with six fluorine atoms comprising F–S bond length of 1.6 Å and F–S–F bond angles 90°. These results are in good agreement with the previous reports [56,57].

Next, we will discuss the optimized structures of the functionalized GQDs both with and without the adsorbed molecules, followed by their electronic properties. For the adsorption process, initially the molecules are placed parallel to the GQDs so as to provide them with more access to the surface areas.

#### 3.1.1 Molecule adsorption on phenalenyl

**Fig. 2:**
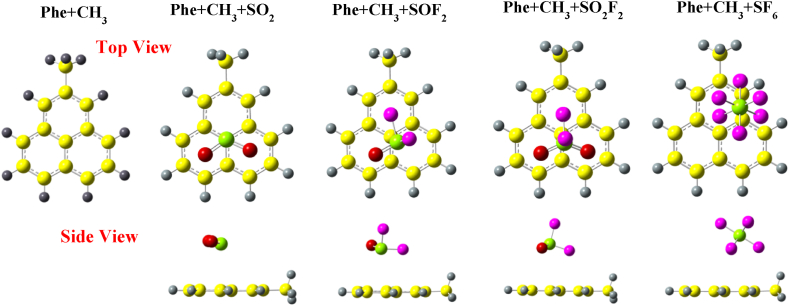
Optimized structures of SO_2_, SOF_2_, SO_2_F_2_, and SF_6_ over CH_3_ edge-functionalized phenalenyl calculated with wB97XD functional.

In Fig. 2, we present the optimized structures of isolated Phe + CH_3_, and the structures with various gas molecules adsorbed on it. For the SO_2_ molecule, it is located slightly towards the centre of phenalenyl with its oxygen atoms over both hexagonal rings with S–C bond distance of 3.1 Å. The SO_2_ structure does not present any noticeable modification in the bond lengths, however, the O–S–O bond angle has decreased from 119.127° to 118.42°. In the case of SOF_2_ adsorption on Phe + CH_3_, the S–C adsorption distance changes from 2 Å to 3.2 Å with no modification in S–O and S–F bond lengths. The bond angle of F–S–O decreases from 107.07° to 106.50° and the bond angle of F–S–F changes from 92.652° to 92.126°. When SO_2_F_2_ is adsorbed on Phe + CH_3_, the S–C adsorption distance becomes 3.5 Å with minor to no change in bond lengths and bond angles of the gas molecule. The SO_2_F_2_ molecule is slightly shifted with fluorine atom tilting towards the carbon atoms. In the case of SF_6_ adsorption on Phe + CH_3_, the S–C adsorption distance is the highest with 4.47 Å, as compared to all other cases considered here. The bond lengths and bond angles of SF_6_ depict no change in its structure as molecule is shifted far away and towards the edge of Phe + CH_3_.

**Fig. 3:**
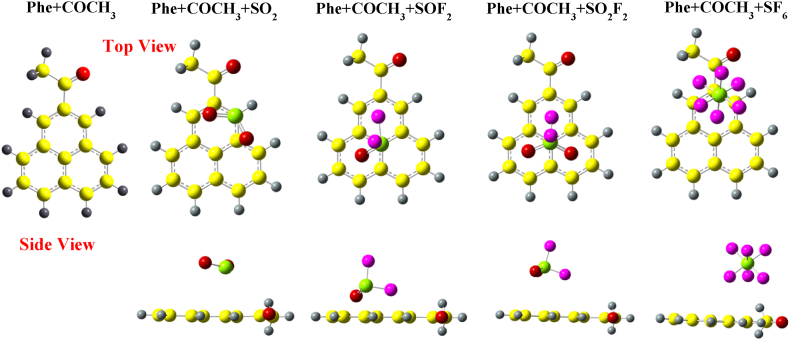
Optimized structures of SO_2_, SOF_2_, SO_2_F_2_, and SF_6_ over COCH_3_ edge-functionalized calculated with wB97XD functional.

In Fig. 3, we present the optimized structure of Phe + COCH_3_ with various gas molecules (SO_2_, SOF_2_, SO_2_F_2_ and SF_6_) adsorbed on it. Similar to the case of SO_2_ adsorption on Phe + CH_3_, the molecule is located slightly towards the edge, closer to the oxygen atom of functional group. The O–S–O bond angle has decreased from 119.127° to 118.35° (lower than Phe + CH_3_). In the case of SOF_2_ adsorption on Phe + COCH_3_, the molecule is slightly shifted with S–C distance 3.2 Å, similar to the case of SOF_2_ over Phe + CH_3_. The bond angle of F–S–O decrease from 107.07° to 106.62°, and bond angle of F–S–F changes from 92.652° to 92.28°. In the case of SO_2_F_2_ molecule over Phe + COCH_3,_ the adsorbed molecule has not been shifted, but the adsorption distance changes to 3.5 Å with fluorine atom facing the GQD. Similar to SF_6_ adsorption over Phe + CH_3_, SF_6_ is attracted towards the functional group making the initial S–C distance of 4.7 Å with no noticeable alteration in bond lengths and bond angles.

**Fig. 4:**
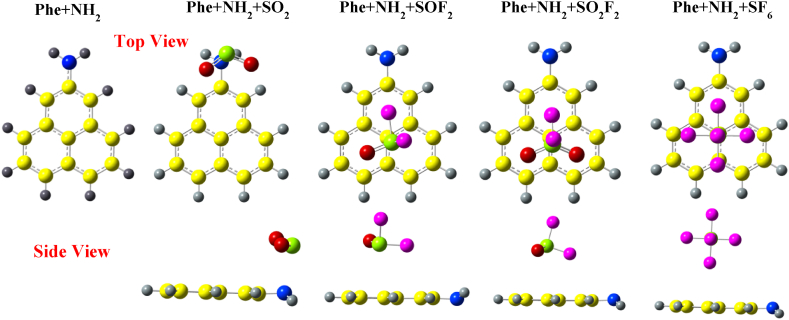
Optimized structures of SO_2_, SOF_2_, SO_2_F_2_, and SF_6_ over NH_2_ edge-functionalized phenalenyl calculated with wB97XD functional.

Fig. 4 shows the optimized structures of gas molecules on Phe + NH_2_. In the case of SO_2_ adsorption, the molecule positions itself slightly towards the edge of the GQD, with the sulfur atom facing the hydrogen atom of –NH_2_ functional group. The adsorption distance between the sulfur and nitrogen atom is 2.62 Å and the O–S–O bond angle has decreased from 119.127° to 117.88°. For the SOF_2_ molecule, the adsorption is similar to that on both –CH_3_ and –COCH_3_ group, but on the opposite side of the Phe + NH_2_ GQD. The bond angle of F–S–O decreases from 107.07° to 106.5° and the bond angle of F–S–F changes from 92.652° to 92.09°. The SO_2_F_2_ molecule adsorbed on Phe + NH_2_ presents similar results as on Phe + CH_3_. In contrast, SF_6_ adsorption on Phe + NH_2_ results in contrasting structural outcomes as compared to the other two functional groups, –CH_3_ and –COCH_3_. The molecule is repelled away from the GQD, with the S–F distance increasing from 2 Å to 2.9 Å.

#### 3.1.2 Molecule adsorption on triangulene

**Fig. 5:**
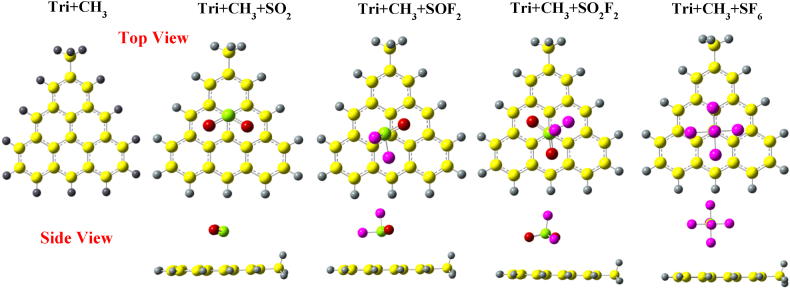
Optimized structures of SO_2_, SOF_2_, SO_2_F_2_, and SF_6_ over CH_3_ edge-functionalized triangulene calculated with wB97XD.

Fig. 5 presents the optimized structures of Tri + CH_3_ with and without gas molecules. The adsorption of SO_2_ on Tri + CH_3_ is similar to that on Phe + CH_3_, with a similar adsorption distance and position after optimization. The bond angles are also analogous to those of the SO_2_ molecule adsorbed on Phe + CH_3_. The SOF_2_ molecule realigns itself to the opposite edge of functional group –CH_3_ with no changes in the S–O and S–F bond lengths. The bond angle of F–S–O decreases from 107.07° to 106.64° and bond angle of F–S–F changes from 92.652° to 92.23°. Similarly, to SOF_2_, SO_2_F_2_ is also repelled from the –CH_3_ functional group to the edges of Tri + CH_3_. In contrast to the SF_6_ adsorption on Phe + CH_3_, SF_6_ on Tri + CH_3_ is moved slightly away from the functional group with the adsorption distance of 2.9 Å. However, similar to SF_6_ on Phe + CH_3_, there are no obvious changes in bond lengths or bond angles.

**Fig. 6:**
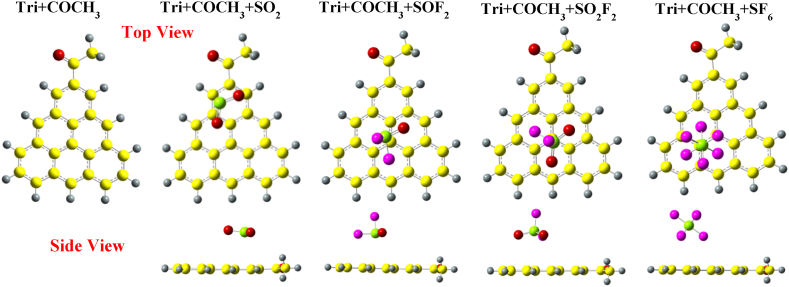
Optimized structures of SO_2_, SOF_2_, SO_2_F_2_, and SF_6_ over COCH_3_ edge-functionalized triangulene calculated with wB97XD.

Fig. 6 presents the optimized structures of Tri + COCH_3_ along with gases. Unlike the adsorption of SO_2_ over Phe + COCH_3_, the molecule positions itself towards the methyl group of Tri + COCH_3_. The O–S–O bond angle has decreased from 119.127° to 118.42°. The structure of SOF_2_ and SO_2_F_2_ are similar to their adsorption on Tri + CH_3_. In contrast to SF_6_ adsorption over Phe + COCH_3_, SF_6_ is repelled away from the system, with a final adsorption distance of 3.9 Å.

**Fig. 7:**
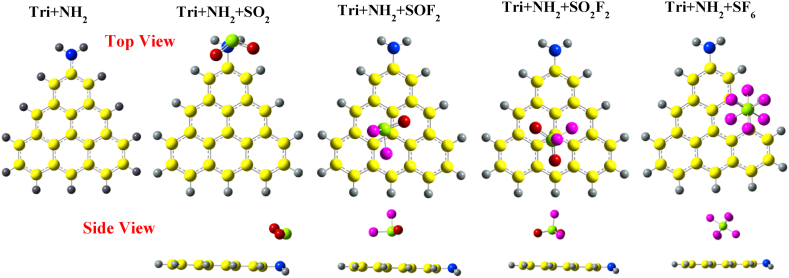
Optimized structures of SO_2_, SOF_2_, SO_2_F_2_, and SF_6_ over NH_2_ edge-functionalized triangulene calculated with wB97XD functional.

Fig. 7 presents the optimized structures of SO_2_, SOF_2_, SO_2_F_2_ and SF_6_ adsorbed over Tri + NH_2_. The sulfur atom of SO_2_ molecule is attracted towards the nitrogen atom of the –NH_2_ group in Tri + NH_2_ with an adsorption distance of 2.62 Å. The O–S–O bond angle has decreased from 119.127° to 117.87°. The structural properties of SOF_2_ are similar to its adsorption on Tri + COCH_3_. Additionally, the structure of SO_2_F_2_ molecule when adsorbed on Tri + NH_2_ is similar to its adsorption on Tri + COCH_3_. The adsorption of SF_6_ on Tri + NH_2_ is similar to its adsorption on Phe + NH_2_, with the SF_6_ molecule slightly attracted towards the edge of Tri + NH2, with an adsorption distance of 4.6 Å.

#### 3.1.3 Molecule adsorption on extended triangulene

**Fig. 8:**
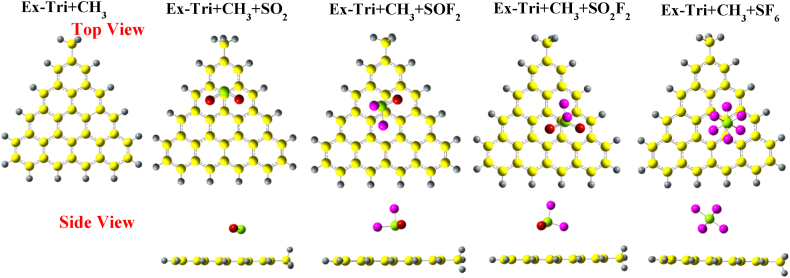
Optimized structures of SO_2_, SOF_2_, SO_2_F_2_, and SF_6_ over CH_3_ edge-functionalized extended-triangulene calculated with wB97XD functional.

Fig. 8 presents the optimized structures of pristine Ex-Tri + CH_3_, along with those with the gas molecules adsorbed on it. For SO_2_, the molecule slightly tilts over the carbon atom, resulting in an S–C bond distance of 3.1 Å, which is similar to the case of Phe + CH_3_. The SO_2_ structure does not lead to any noticeable changes in the bond lengths, but the O–S–O bond angle decreases from 119.127° to 118.48°. SOF_2_ adsorption over Ex-Tri + CH_3_ is similar to that of SOF_2_ over Tri + CH_3_.

**Fig. 9:**
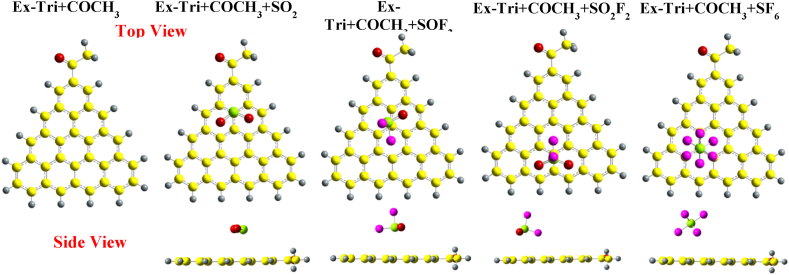
Optimized structures of SO_2_, SOF_2_, SO_2_F_2_, and SF_6_ over COCH_3_ edge-functionalized extended-triangulene calculated with wB97XD.

When SO_2_F_2_ is adsorbed over Ex-Tri + CH_3_, the S–C adsorption distance becomes 3.4 Å with minor to no change in bond lengths and bond angles of the gas molecule. Analogous to Phe + CH_3_, the SO_2_F_2_ molecule slightly shifts towards the edges of Ex-Tri + CH_3_. The SF_6_ adsorption on Ex-Tri + CH_3_ results in a similar structure as in the case of Phe + CH_3_. Similar adsorption properties are observed when SO_2_ and SOF_2_ are adsorbed over Ex-Tri + COCH_3_ (See Fig. 9). However, both SO_2_F_2_ and SF_6_ are shifted towards the edge of GQD after adsorption, with similar adsorption distances as in case of Ex-Tri + CH_3_.

**Fig. 10:**
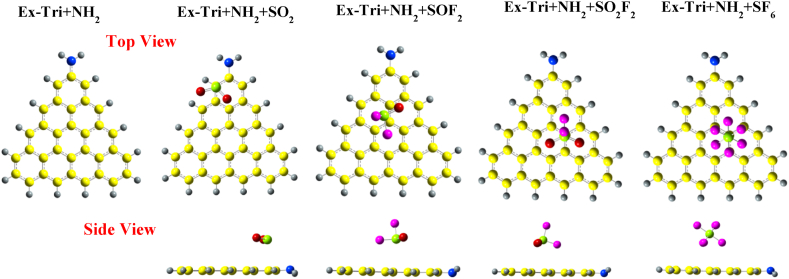
Optimized structures of SO_2_, SOF_2_, SO_2_F_2_, and SF_6_ over NH_2_ edge-functionalized extended-triangulene calculated with wB97XD functional.

Fig. 10 presents the optimized structures of SO_2_, SOF_2_, SO_2_F_2_ and SF_6_ adsorption over Ex-Tri + NH_2_. SO_2_ is attracted towards the NH_2_ functional group with adsorption distance between S and C atom 3.01 Å. The adsorption of SOF_2_, SO_2_F_2_ and SF_6_ results in similar configurations except for the adsorption distances, which, for the three molecules become 3.19 Å, 3.4 Å, and 3.9 Å, respectively.

To confirm the thermodynamical stability, we have calculated the formation energy (E_f_) using Eq. (3) for functionalized GQDs. The E_f_ for phenalenyl functionalized with –CH_3_, –COCH_3_ and –NH_2_ are −6.314 eV, −6.957 eV and −6.388 eV, respectively. While in case of E_f_ for triangulene functionalized with –CH_3_, –COCH_3_ and –NH_2_ are −6.729 eV, −7.153 eV and −6.79 eV, respectively. Further, E_f_ for extended triangulene functionalized with –CH_3_, –COCH_3_ and –NH_2_ are −7.029 eV, −7.072 eV and −7.090 eV, respectively. The negative E_f_ for all GQDs confirms their thermodynamical stability and possible fabrication experimentally. Raman calculation is a powerful analytical tool for characterizing the structural stability of materials. The Raman spectrum is sensitive to the vibrational modes of a material and can provide information about the chemical bond structure and symmetry of a material. By monitoring changes in the Raman plot, researchers can determine if a material is undergoing structural changes or if its structure is remaining stable. The Raman plot can be used as a measure of the stability of the structure of a material over time. Raman analysis (presented in Fig. S8 of Supplementary material) suggests that all the systems are dynamically stable as there are no imaginary frequencies.

### 3.2 Electronic and adsorption properties

Calculations regarding the sensing ability are made using the principle that as the adsorbed gas molecules interact with the GQDs, the electron distribution in the system must change. This electron reorganization should manifest as a measurable change in the ability to conduct an electrical current, known as conductivity. The fundamental parameter that can be calculated using this is the molecular orbits or HOMO-LUMO (H-L) gap. The electron donating and accepting ability of a system can be defined using the value of HOMO and LUMO energy. These molecular orbitals play vital role in electronic and optical properties, luminescence, photochemical reaction, UV-VIS, quantum chemistry etc. The molecular orbitals help in predicting the most reactive position of the studied system. Further, Mulliken charge transfer is calculated and tabulated in Table 1, Table 2, Table 3, confirming the sensitivity of gas molecules SO_2_, SOF_2_, SO_2_F_2_ and SF_6_ over phenalenyl, triangulene and extended triangulene.

**Table 1:** Energy H-L gap (E_g_), Adsorption Energy (E_ad_) and Recovery time (τ) of structure of SO_2_, SOF_2_, SO_2_F_2_, and SF_6_ adsorption on phenalenyl.

Structure	Charge (e)	E_g_ (eV)		E_ad_ (eV)		τ (ps)	
		B3LYP	ωB97XD	B3LYP	ωB97XD	B3LYP	ωB97XD
Phe + CH_3_	–	3.913	7.511	–	–	–	–
Phe + CH_3_ + SO_2_	+0.03462	3.595	7.544	−0.124	−0.256	121	19,988
Phe + CH_3_ + SOF_2_	+0.02214	3.907	7.516	−0.137	−0.268	200	31,796
Phe + CH_3_ + SO_2_F_2_	+0.00311	3.901	7.537	−0.066	−0.227	12	6509
Phe + CH_3_ + SF_6_	−0.00998	3.696	7.502	−0.074	−0.184	17	1233
Phe + COCH_3_	–	4.083	7.71	–	–	–	–
Phe + COCH_3_ + SO_2_	+0.04581	4.108	7.40	−0.26	−0.241	23,332	11,188
Phe + COCH_3_ + SOF_2_	+0.01746	3.956	7.71	−0.128	−0.252	141	17,121
Phe + COCH_3_ + SO_2_F_2_	+0.00183	4.041	7.724	−0.064	−0.223	11	5576
Phe + COCH_3_ + SF_6_	−0.01299	4.08	7.714	−0.032	−0.201	3	2381
Phe + NH_2_	–	2.847	6.38	–	–	–	–
Phe + NH_2_ + SO_2_	+0.09231	3.359	7.01	−0.37	−0.429	1,644,093	16,112,404
Phe + NH_2_ + SOF_2_	+0.02352	2.924	6.562	−0.247	−0.33	14,110	349,899
Phe + NH_2_ + SO_2_F_2_	+0.00294	3.002	6.62	−0.122	−0.28	112	50,579
Phe + NH_2_ + SF_6_	−0.0028	2.549	6.58	−0.141	−0.166	233	614

**Table 2:** Energy H-L gap (E_g_), Adsorption Energy (E_ad_) and Recovery time (τ) of structure of SO_2_, SOF_2_, SO_2_F_2_, and SF_6_ adsorption on triangulene.

Structure	Charge(e)	E_g_ (eV)		E_ad_ (eV)		τ (ps)	
		B3LYP	ωB97XD	B3LYP	ωB97XD	B3LYP	ωB97XD
Tri + CH_3_	–	3.669	7.182	–	–	–	–
Tri + CH_3_ + SO_2_	+0.03745	3.23	7.025	−0.119	−0.261	99	24,253
Tri + CH_3_ + SOF_2_	+0.01667	3.62	7.192	−0.111	−0.282	73	54,644
Tri + CH_3_ + SO_2_F_2_	+0.00028	3.653	7.190	−0.065	−0.257	12	20,774
Tri + CH_3_ + SF_6_	−0.00356	3.668	7.180	−0.021	−0.145	2	272
Tri + COCH_3_	–	3.734	7.267	–	–	–	–
Tri + COCH_3_ + SO_2_	+0.03396	3.261	6.93	−0.109	−0.247	67	14,110
Tri + COCH_3_ + SOF_2_	+0.0145	3.698	7.301	−0.127	−0.275	136	41,680
Tri + COCH_3_ + SO_2_F_2_	−0.0048	3.72	7.288	−0.061	−0.261	10	24,253
Tri + COCH_3_ + SF_6_	−0.01106	3.73	7.265	−0.03	−0.243	3	12,088
Tri + NH_2_	–	2.808	6.312	–	–	–	–
Tri + NH_2_ + SO_2_	+0.09266	3.275	6.853	−0.365	−0.427	1,354,983	14,912,345
Tri + NH_2_ + SOF_2_	+0.01645	2.918	6.488	−0.177	−0.335	940	424,556
Tri + NH_2_ + SO_2_F_2_	+0.00027	3.017	6.504	−0.162	−0.389	526	3,428,728
Tri + NH_2_ + SF_6_	−0.00811	2.973	6.493	−0.09	−0.254	32	18,499

**Table 3:** Energy H-L gap (E_g_), Adsorption Energy (E_ad_) and Recovery time (τ) of structure of SO_2_, SOF_2_, SO_2_F_2_, and SF_6_ adsorption on extended triangulene.

Structure	Charge (e)	E_g_ (eV)		E_ad_ (eV)		τ (ps)	
		B3LYP	ωB97XD	B3LYP	ωB97XD	B3LYP	ωB97XD
Ex-Tri + CH_3_	–	1.342	4.566	–	–	–	–
Ex-Tri + CH_3_ + SO_2_	+0.04094	1.329	4.432	−0.180	−0.261	1055	24,253
Ex-Tri + CH_3_ + SOF_2_	+0.01486	1.367	4.527	−0.133	−0.285	171	61,371
Ex-Tri + CH_3_ + SO_2_F_2_	−0.00064	1.429	4.555	−0.102	−0.260	51	23,332
Ex-Tri + CH_3_ + SF_6_	−0.01252	1.404	4.570	−0.039	−0.256	4	19,988
Ex-Tri + COCH_3_	–	1.386	4.544	–	–	–	–
Ex-Tri + COCH_3_ + SO_2_	+0.0358	1.183	4.407	−0.142	−0.246	242	13,575
Ex-Tri + COCH_3_ + SOF_2_	+0.01236	1.448	4.520	−0.077	−0.273	19	38,580
Ex-Tri + COCH_3_ + SO_2_F_2_	−0.00098	1.382	4.503	−0.042	−0.262	5	25,210
Ex-Tri + COCH_3_ + SF_6_	−0.01315	1.401	4.543	−0.034	−0.261	3	24,253
Ex-Tri + NH_2_	–	2.767	6.22	–	–	–	–
Ex-Tri + NH_2_ + SO_2_	+0.05453	2.491	6.215	−0.23	−0.320	7310	237,660
Ex-Tri + NH_2_ + SOF_2_	+0.01644	2.873	6.395	−0.219	−0.344	4777	601,390
Ex-Tri + NH_2_ + SO_2_F_2_	−0.000093	2.968	6.405	−0.161	−0.324	506	277,423
Ex-Tri + NH_2_ + SF_6_	−0.01191	2.927	6.394	−0.067	−0.324	13	277,423

**Fig. 11:**
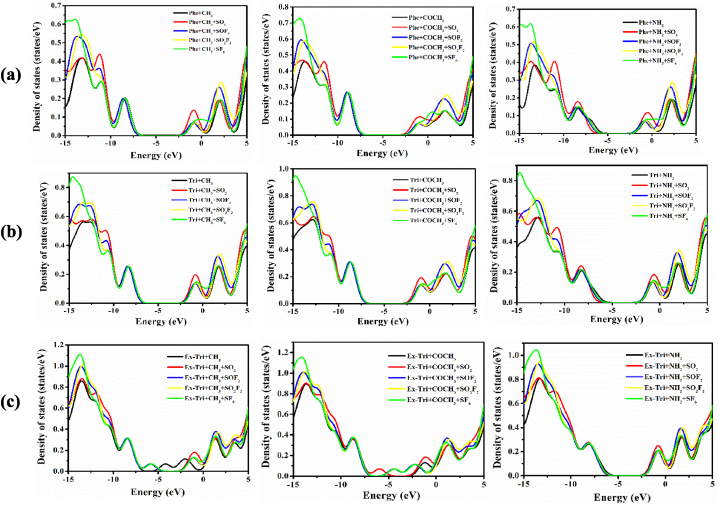
Density of states of adsorption of SO_2_, SOF_2_, SO_2_F_2_, and SF_6_ with (a) phenalenyl (b) triangulene and (c) extended-triangulene systems with wB97XD functional.

Fig. 11(a–c) depicts total density of states (TDOS) to further analyse the electronic structure from the perspective of size of the GQDs (phenalenyl, triangulene and extended triangulene), functional groups, and adsorbed molecules SO_2_, SOF_2_, SO_2_F_2_ and SF_6_. The total density of states gives the number of states present per energy range. The discrete levels are modified using Gaussian $\frac{1}{\sqrt{2\pi }\alpha }\exp\left[-\frac{{\left(\varepsilon -\varepsilon_{i}\right)}^{2}}{2\alpha^{2}}\right]$ as the broadening function. The adsorption of these gas molecules on Phe + CH_3_ leads to minor changes in highest occupied molecular orbitals (HOMO) and lowest unoccupied molecular orbitals (LUMO). However, the highest peak of Phe + CH_3_ located around −13 eV in valence band shifts and even splits in case of Phe + CH_3_ + SF_6_. The HOMO and LUMO in Phe + CH_3_ + SOF_2_ and Phe + CH_3_ + SO_2_F_2_ depict minor changes attributable to slight alteration in their structural properties. The energy HOMO-LUMO gap (H-L gap) of all considered systems are presented in Table 1.

The DOS plots of the gas molecules adsorbed on Phe + COCH_3_ show that the HOMO exhibit trivial difference in all cases except Phe + COCH_3_ + SO_2_. However, the LUMOs are shifted slightly towards higher energies. The molecules adsorbed over Phe + NH_2_ largely show similar features except that a shift in HOMO is observed for the case of Phe + NH_2_ + SO_2_. In all three cases of functionalized phenalenyl, SF_6_ adsorbed systems acquire the highest peak in the valence region. The H-L gap changes significantly in case of Phe + NH_2_ as compared to others.

The middle row in Fig. 11 shows the results of DOS for triangulene functionalized systems. The energy gap changes noticeably only in case of Tri + CH_3_+SO_2_, from 7.18 eV to 7.02 eV. The minimum change in the gap is observed in case of Tri + CH_3_+SF_6_. Similar changes are also seen also in SO_2_, SOF_2_, SO_2_F_2_ and SF_6_ adsorbed Tri + COCH_3_ systems. In the case of Tri + NH_2_, a drastic modification is observed when SO_2_ is adsorbed, leading to a change in the energy H-L gap from 6.31 eV to 6.85 eV. The minimum H-L gap change is observed in SOF_2_ adsorbed systems. The energy H-L gap values of Ex-Triangulene are presented in Table 3 with B3LYP and ωB97XD. The largest H-L gap is found for Ex-Tri + NH_2_ among all functionalized GQDs. The ωB97XD (long range corrected) predicted energy gaps are considerably larger than the B3LYP predicted gap values. This is mainly due to the inclusion of full Hartree–Fock exchange term in the ωB97XD functional at long distances [58]. It is concluded that for electronic properties such as the H-L gaps, B3LYP shows better results; however, ωB97XD shows better results for studying the adsorption mechanism. The sizes and functionalization of GQDs strongly influence its structural and electronic properties. The energy gap value is a crucial parameter in defining the electrical conductivity (σ) of materials because the energy required to take out an electron from the outer shell to become a free portable charge carrier is proportional to the H-L gap. The link between the E_g_ and σ of a material can be mathematically represented by the following formula [59]:

(4) $$\sigma \propto e^{\frac{{-E}_{g}}{2kT}}$$

where k and T are the Boltzmann's constant and the temperature respectively. The conductivity of a material is inversely proportional to its H-L gap, as shown by Eq. (4). Therefore, a smaller H-L gap value leads to higher σ at a given temperature T. Consequently, when gas molecules are adsorbed onto the surfaces of graphene quantum dots, a significant decrease in their H-L gap value leads to an increase in their conductivity. The reduction of E_g_ is observed in case of Phe + CH_3_+SF_6,_ Phe + COCH_3_ + SO_2_, Tri + CH_3_ + SO_2,_ Tri + COCH_3_ + SO_2_, Ex-Tri + CH_3_ + SO_2_ and Ex-Tri + COCH_3_ + SO_2_. As a result, the conductivity of the functionalized GQDs increases, providing evidence of the robust interaction between the adsorbed gas molecule and the GQDs. This alteration in the molecular orbitals of the GQDs because of the adsorbed molecules could be identified electronically, which suggests its potential application in sensor technology. The adsorption energies calculated through Eq. (2) for all optimized systems are given in Table 1, Table 2, Table 3. The gas molecules get adsorbed on the energetically stable structures of functionalized GQDs, typically at distances in the range 2.46–4.47 Å. These considerably large adsorption distances prohibit the formation of chemical bonds, resulting in physisorption [[60], [61], [62], [63], [64]].

**Fig. 12:**
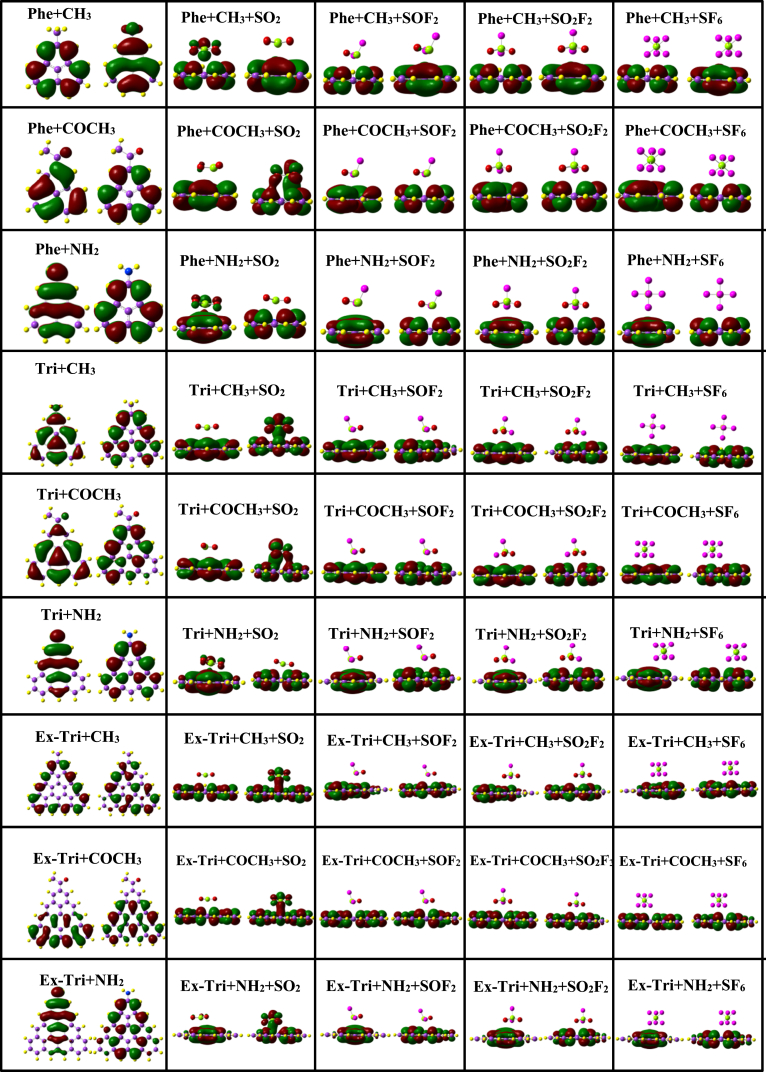
HOMO-LUMO plot of adsorption of SO_2_, SOF_2_, SO_2_F_2_, and SF_6_ with phenalenyl, triangulene and extended-triangulene systems with wB97XD functional.

The adsorption energy (E_ad_) calculated for SO_2_, SOF_2_, SO_2_F_2_ and SF_6_ over Phe + CH_3_ is −0.256 eV, −0.268 eV, −0.227 eV and −0.184 eV respectively. The lower adsorption energy in SO_2_F_2_ and SF_6_ can be attributed to the relatively larger adsorption distances. In Phe + COCH_3_, the highest adsorption energy is observed for the sensing of SOF_2_ with the value −0.252 eV. Of all the gas molecules adsorbed on various functionalized GQDs, the largest adsorption energy of −0.429 eV is achieved for the case of SO_2_ over Phe + NH_2._ The adsorption energies of functionalized triangulene for various gas molecules are presented in Table 2. For sensing of SO_2_, Tri + NH_2_ (−0.427 eV) provides superior adsorption energy as compared to Tri + CH_3_ (−0.261 eV) and Tri + COCH_3_ (−0.247 eV). Similarly, Tri + NH_2_ is energetically better for sensing of SOF_2_, SO_2_F_2_ and SF_6_ in comparison with other triangulene functionalizations. In Table 3, we present the adsorption energies of all the functionalized GQDs for various gas molecules. It is obvious from the table that for GQDs functionalized with the groups CH_3_, COCH_3_ and NH_2_, SOF_2_ shows high adsorption energies −0.285 eV, −0.273 eV, and −0.344 eV, respectively. To understand the electronic interaction between the gas molecules and functionalized GQDs, we plot in Fig. 12 the frontier molecular orbitals.

It is clear from the figure that SOF_2_, SO_2_F_2_ and SF_6_ molecules do not contribute to the formation of either HOMO or LUMO. Instead, these orbitals are completely confined to the surfaces of the functionalized GQDs, confirming the physisorption character of the adsorption processes. Furthermore, the H-L gap of SOF_2_, SO_2_F_2_ and SF_6_ over the functionalized GQDs is close to the corresponding pristine values (see Table 1, Table 2, Table 3). In contrast, the behaviour of SO_2_ on the functionalized GQDs is qualitatively different (as shown in Fig. 12). The HOMO (in the case of Phe + CH_3_, Phe + COCH_3_, Phe + NH_2_, Tri + NH_2_) and LUMO (in the case of Tri + CH_3_, Tri + COCH_3_, Ex-Tri + CH_3_, Ex-Tri + COCH_3_, Ex-Tri + NH_2_) are shared indicating strong hybridization.

**Fig. 13:**
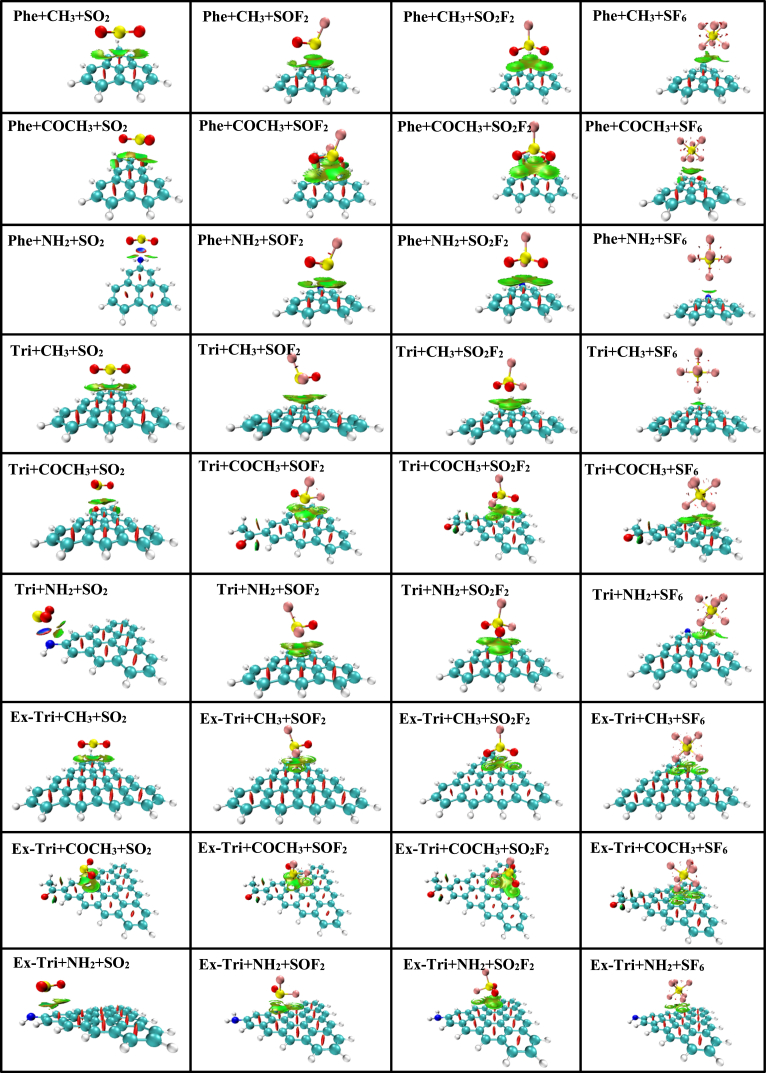
NCI plots with wB97XD functional. Blue represents strong attractive interactions, green indicates interactions and red indicates repulsive/steric interactions. (For interpretation of the references to colour in this figure legend, the reader is referred to the Web version of this article.)

As a result, the H-L gap is altered for these cases. Additionally, non-covalent interaction (NCI) analyses were performed using Multiwfn [64] and VMD [65] packages at the same level of theory to account for H-bonds and non-covalent interactions and are presented in Fig. 13. The strong attraction and repulsion are represented by blue and red region respectively. The van der Waals interaction is shown by the green region. The coloration of the isosurfaces is determined by the values of sign (λ_2_)ρ (a.u.), ranging from 0.03 to 0.02 a. u. It is observed that strong attractions (blue region) are found in SO_2_ over NH_2_ functionalized phenalenyl and triangulene. Steric effect or strong repulsion is found between the hexagonal rings in GQDs, however, van der Waals interaction were observed between the gas molecules and GQDs systems.

### 3.3 Recovery time

The recovery characteristics of a gas sensor are crucial for determining its usefulness and reusability. The traditional method for evaluating recovery time is by heating the substrate, however, strong chemical reactions or chemisorption between gases and the substrate can result in long recovery times. Theoretical calculations to determine recovery time (τ) can be performed using transition state theory and the Van't-Hoff-Arrhenius expression [66,67], resulting in the expression:

(5) $$\tau =\upsilon^{-1}e^{-E_{ad}/KT}$$

Here, υ is defined as the attempt frequency, K and T denote Boltzmann constant (∼8.318∗10^−3^ kJ/mol K), and temperature respectively. In the present cases, 10^12^ s^−1^ of attempt frequency and 298 K temperature is taken for the recovery time calculation [68,69].

The van der Waals interactions between functionalized GQDs and gas molecules have a significant impact on the recovery time of a gas sensor. This is because van der Waals interactions can strongly influence the adsorption and desorption of gas molecules on the surface of the GQDs. The strength of these interactions can determine the amount of energy required for a gas molecule to adsorb or desorb from the surface, which in turn can affect the recovery time of the sensor. Additionally, the van der Waals interactions can also contribute to a shift in the adsorption energy, which can further impact the performance of the sensor. Therefore, understanding and controlling the van der Waals interactions between functionalized GQDs and gas molecules is crucial for optimizing the performance of gas sensors. Comparing the –CH_3_, –COCH_3_ and –NH_2_ functionalized GQDs according to recovery time, the gas molecules are challenging to desorb from Phe + NH_2_, Tri + NH_2_ and Ex-Tri + NH_2_. The recovery times calculated through Eq. (5) are given in Table 1, Table 2, Table 3. In Phe + CH_3_, SO_2_F_2_ and SF_6_ have lower recovery times of 6509 ps and 1233 ps, resulting in better desorption property. Similarly for Phe + COCH_3_, SO_2_F_2_ and SF_6_ have lower recovery time of 5576 ps and 2381 ps. In summary, both Phe + CH_3_ and Phe + COCH_3_ have superior recovery properties and therefore perform better at lower temperatures as compared to Phe + NH_2_. For sensing SO_2_ and SOF_2_, Phe + NH_2_ and Phe + COCH_3_ are not very suitable as they require relatively high working temperature for desorption. Similarly to –NH_2_ functionalized phenalenyl system, Tri + NH_2_ and Ex-Tri + NH_2_ also have high recovery times for SO_2_, SOF_2_, SO_2_F_2_ and SF_6_ gases making them unsuitable for use. In order to put the performance of functionalized GQDs in perspective, we briefly discuss the recovery times of a few other adsorbing materials for the same gas molecules. The α-arsenene with SO_2_, SOF_2_ and SO_2_F_2_ shows recovery times of 0.23s, 0.68 ms and 0.44μs, however β-arsenene with SO_2_, SOF_2_ and SO_2_F_2_ provides 3.65 μs, 0.33 μs and 0.05 μs of recovery times, respectively [70]. The SO_2_, SOF_2_ and SO_2_F_2_ have long recovery times of 400 s, 669 μs and 5.9 ns at 298 K, respectively for Ni-BNNT system [71]. It is found that in Pt–MoS_2_ and Au–MoS_2_, SO_2_ and SOF_2_ are difficult to desorb unless increasing the working temperature [72]. These recovery times are higher as compared to GQDs (in ps) in the present study leading to their superiority and high detection limit.

## 4 Conclusions

In this study, we employed density functional theory (DFT) to investigate the adsorption behaviour and gas sensing properties of size and edge-functionalized graphene quantum dots (GQDs), specifically phenalenyl, triangulene, and extended triangulene. We analyzed properties such as adsorption energy, adsorption distance, recovery time, and density of states. Our results show that functionalization of phenalenyl, triangulene, and extended triangulene alters their electronic properties. However, the adsorption of SO_2_, SOF_2_, SO_2_F_2_, and SF_6_ gases on Phe + CH_3_ leads to minor changes in HOMO and LUMO. A significant change is observed when SO_2_ is adsorbed on –NH_2_ functionalized triangulene, resulting in a change in the energy gap. Furthermore, –NH_2_ functionalized phenalenyl, triangulene, and extended triangulene show superior adsorption energies for SO_2_ sensing compared to other functionalizations. In terms of recovery time, it is challenging to desorb the gases (SO_2_, SOF_2_, SO_2_F_2_, and SF_6_) from Phe + NH_2_, Tri + NH_2_, and Ex-Tri + NH_2_ when using –CH_3_, –COCH_3_, and –NH_2_ functionalized phenalenyl and triangulene. In conclusion, this study offers a microscopic understanding of the ultrafast recovery times of GQDs and their potential applications in sensing toxic environmental gases.

## Author contribution statement

Vaishali Roondhe; Basant Roondhe: Conceived and designed the experiments; Performed the experiments; Analyzed and interpreted the data; Wrote the paper.

Sumit Saxena; Rajeev Ahuja; Alok Shukla: Analyzed and interpreted the data; Contributed reagents, materials, analysis tools or data.

## Data availability statement

Data will be made available on request.

## Declaration of competing interest

The authors declare that they have no known competing financial interests or personal relationships that could have appeared to influence the work reported in this paper.
