# Supplementary material for: On using non-Kekulé triangular graphene quantum dots for scavenging hazardous sulfur hexafluoride components
Source: Heliyon. 2023 Apr 7;9(4):e15388. doi: 10.1016/j.heliyon.2023.e15388 (PMC10130882; doi:10.1016/j.heliyon.2023.e15388)
Supplement: Multimedia component 1 [file mmc1.docx]

**On Using Non-Kekulé Triangular Graphene Quantum Dots for Scavenging Hazardous Sulfur Hexafluoride Components**

**Vaishali Roondhe^1,*^, Basant Roondhe^2^, Sumit Saxena^2^, Rajeev Ahuja^3,4,*^ and Alok Shukla^1,^^[[1]](#footnote-1)^**

^1^Department of Physics, Indian Institute of Technology Bombay, Mumbai-400076, Maharashtra, India

^2^Department of Metallurgical Engineering and Materials Science, Indian Institute of

Technology Bombay, Mumbai-400076, Maharashtra, India

^3^Materials Theory Division, Department of Physics and Astronomy, Uppsala University, Box 516, Uppsala 75120, Sweden

^4^Department of Physics, Indian Institute of Technology Ropar-140001, Punjab, India

**Supplementary Figures**

**Top View**

**Phe + CH_3_ + SO_2_**

**Phe+ CH_3_ + SOF_2_**

**Phe+ CH_3_ +SO_2_F_2_**

**Phe+ CH_3_ + SF_6_**


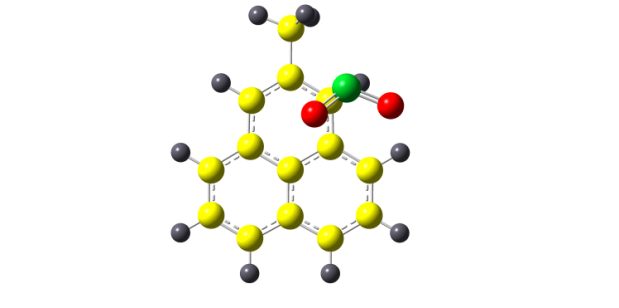

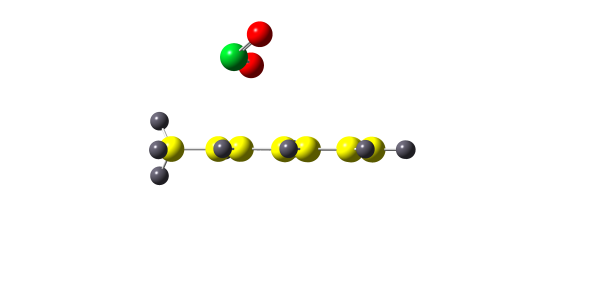

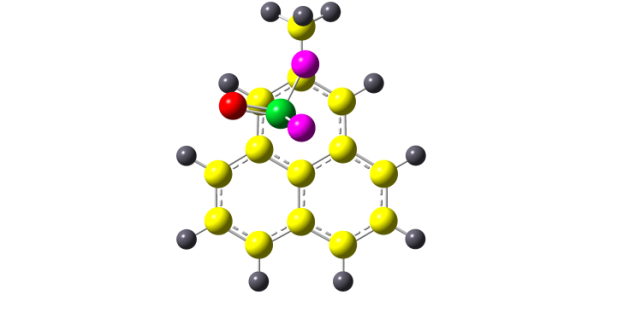

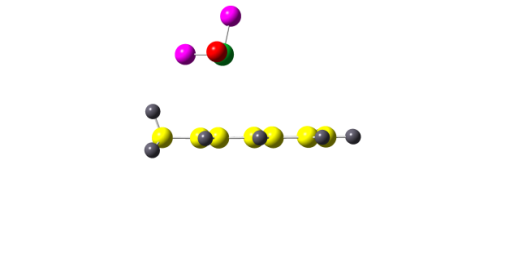

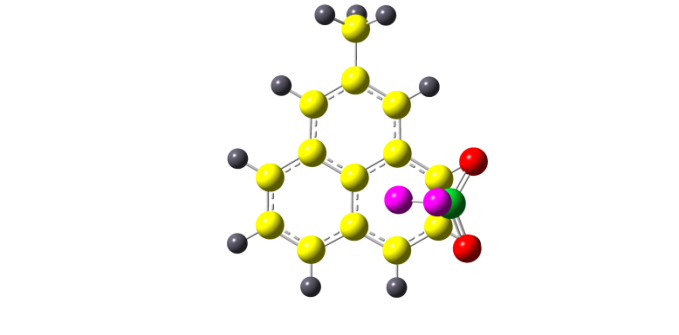

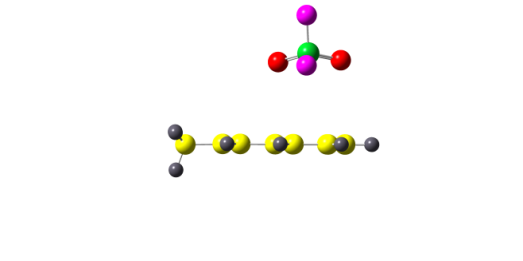

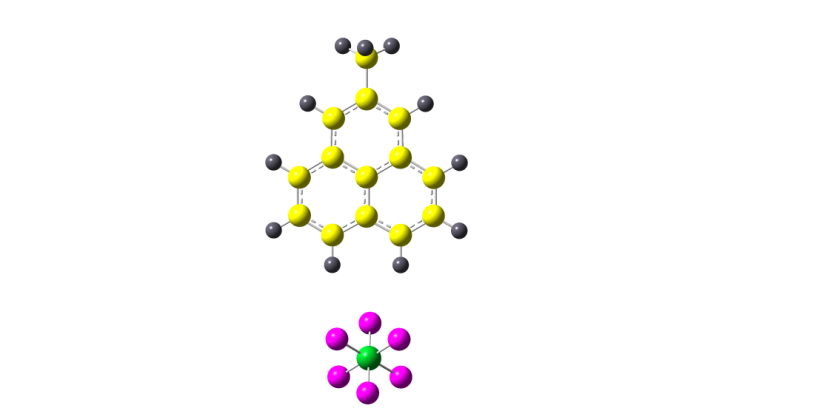

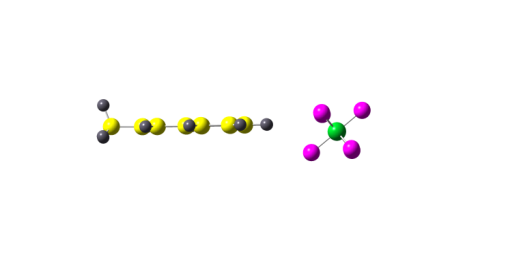


**Phe + CH_3_**


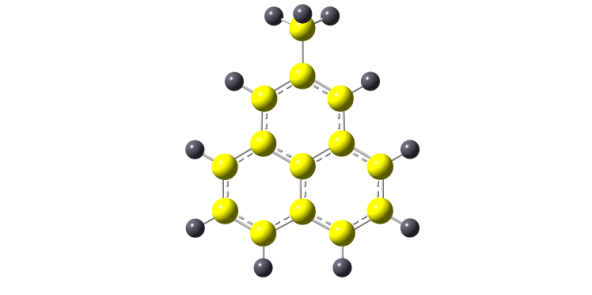


**Side View**

Figure S1: Optimized structures of SO_2_, SOF_2_, SO_2_F_2_, and SF_6_ with CH_3_ edge-functionalized phenalenyl with B3LYP functional. The yellow, grey, green, red and purple balls represent carbon, hydrogen, sulfur, oxygen and fluorine atoms respectively.

**Phe + COCH_3_ + SO_2_**

**Phe+ COCH_3_+ SOF_2_**

**Phe + COCH_3_+ SO_2_F_2_**

**Phe + COCH_3_ + SF_6_**


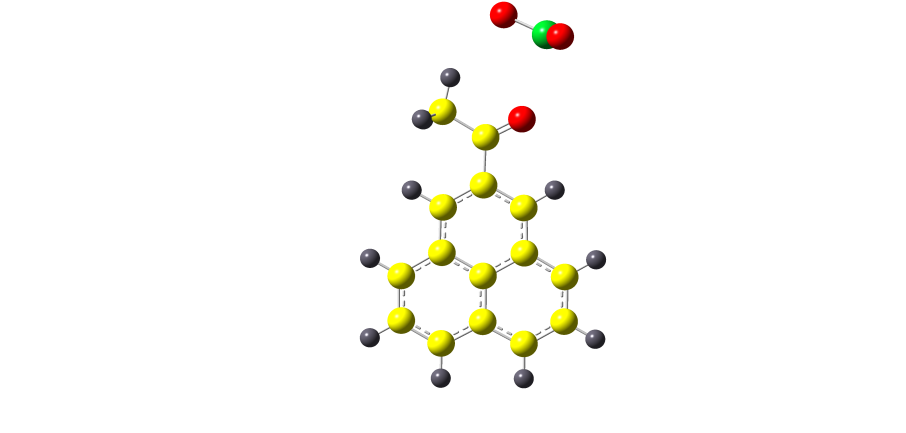

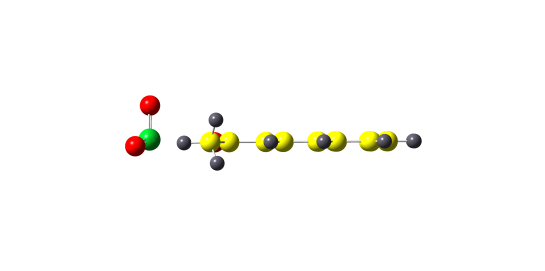

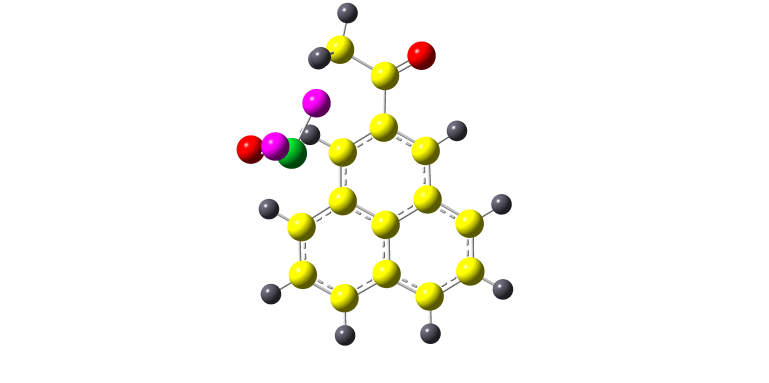

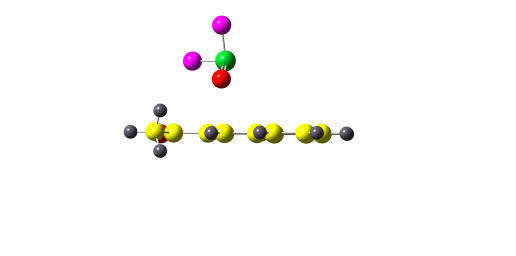

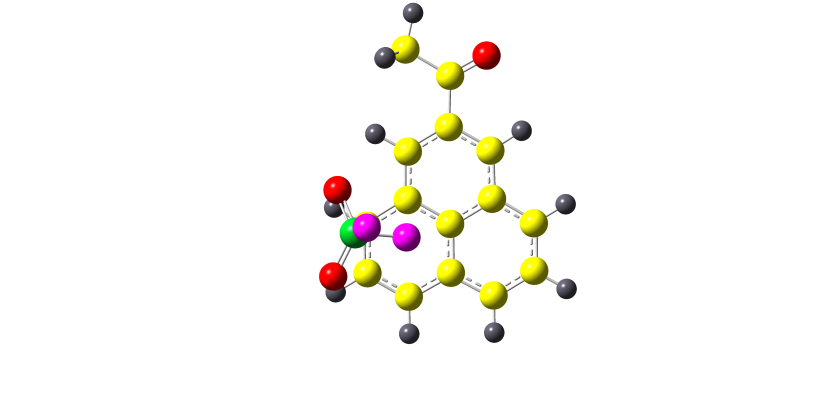

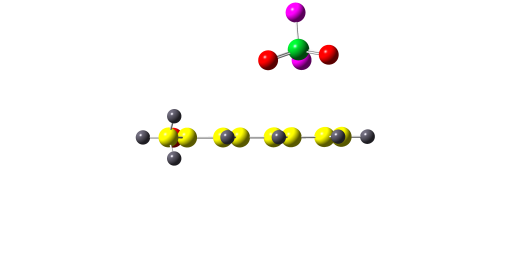

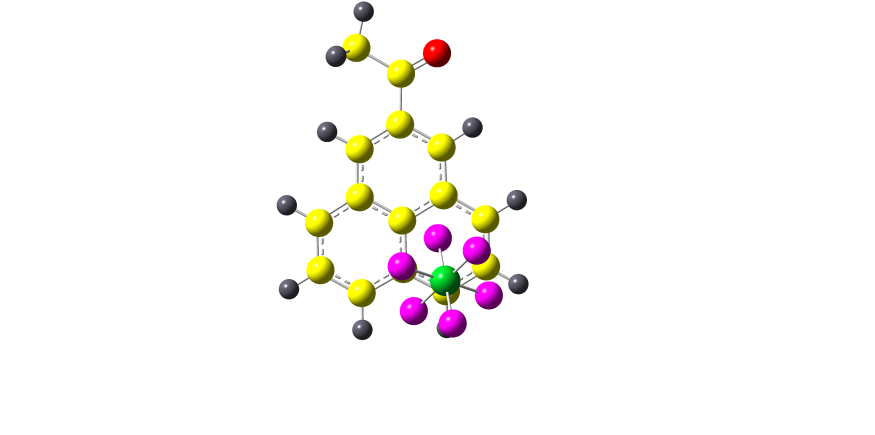

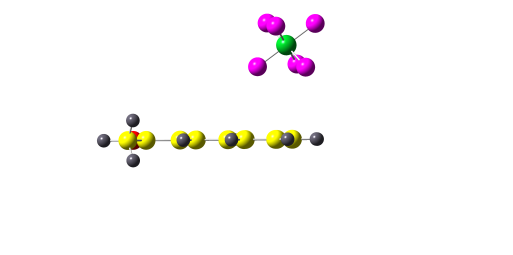


**Phe + COCH_3_**


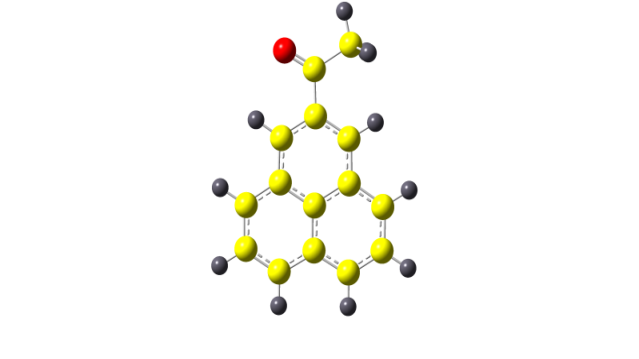


**Top View**

**Side View**

Figure S2: Optimized structures of SO_2_, SOF_2_, SO_2_F_2_, and SF_6_ with COCH_3_ edge-functionalized phenalenyl with B3LYP functional.

**Phe+ NH_2_ + SO_2_**

**Phe+ NH_2_ + SOF_2_**

**Phe + NH_2_ + SO_2_F_2_**

**Phe + NH_2_ + SF_6_**


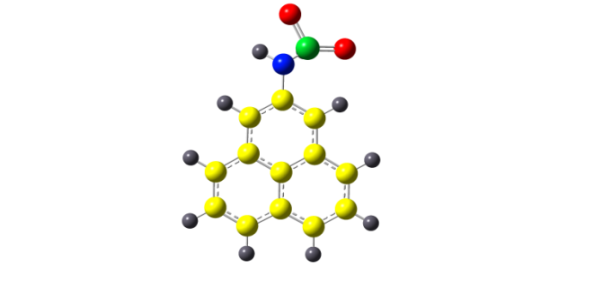

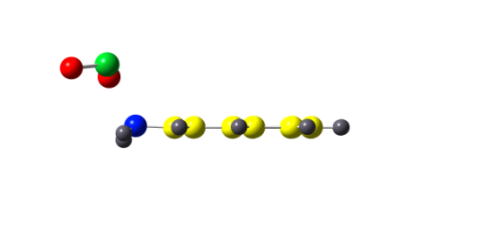

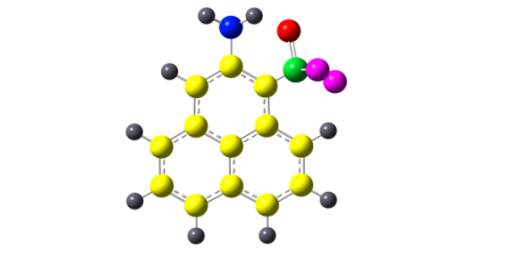

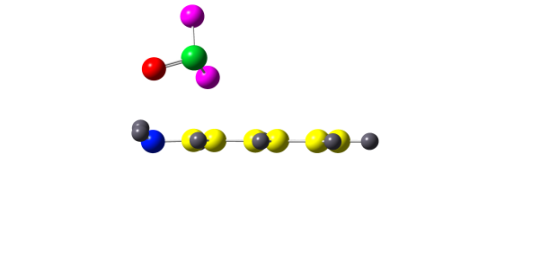

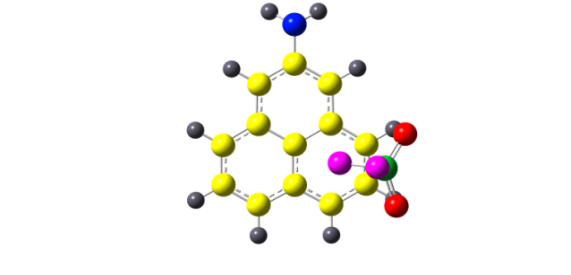

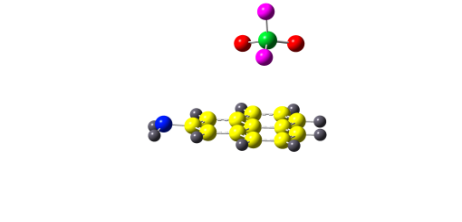

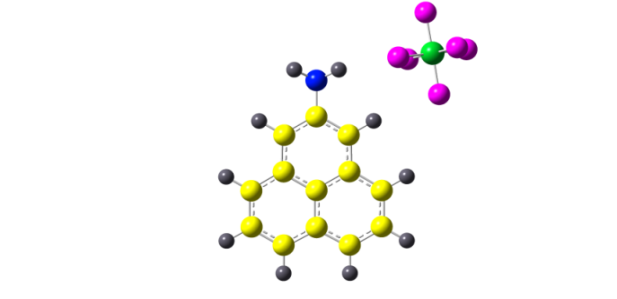

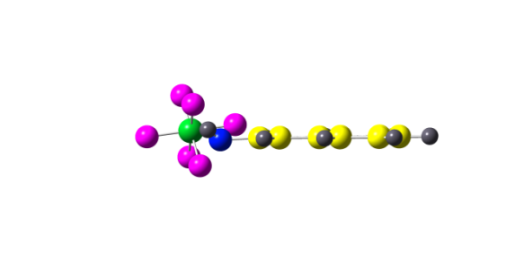


**Phe + NH_2_**


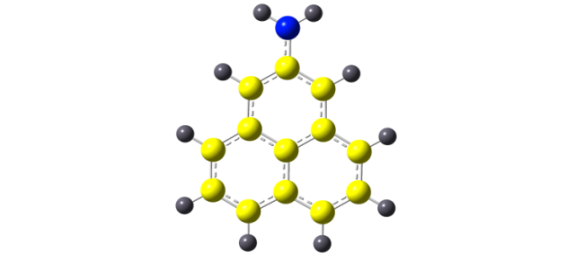


**Top View**

**Side View**

Figure S3: Initial structures of SO_2_, SOF_2_, SO_2_F_2_, and SF_6_ with NH_2_ edge-functionalized phenalenyl with B3LYP functional.

**Tri + CH_3_ + SO_2_**

**Tri + CH_3_ + SOF_2_**

**Tri + CH_3_ + SO_2_F_2_**

**Tri + CH_3_ + SF_6_**


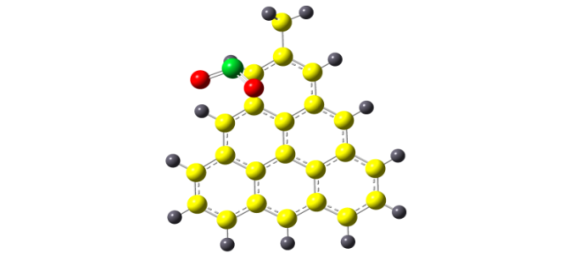

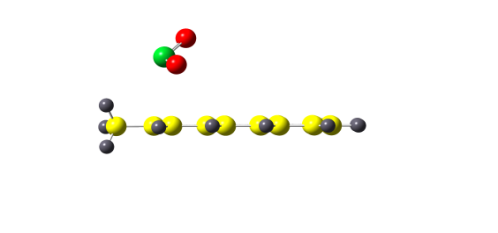

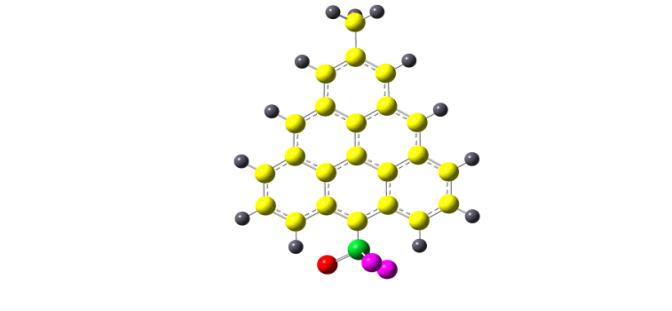

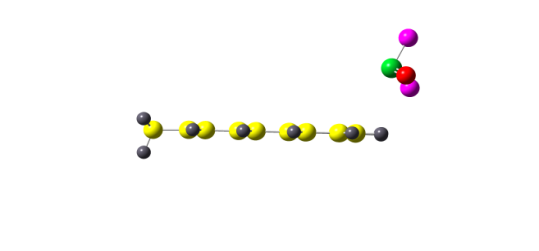

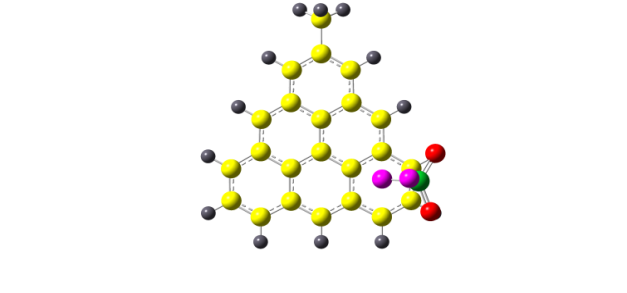

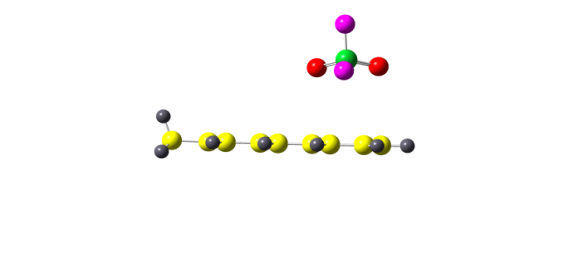

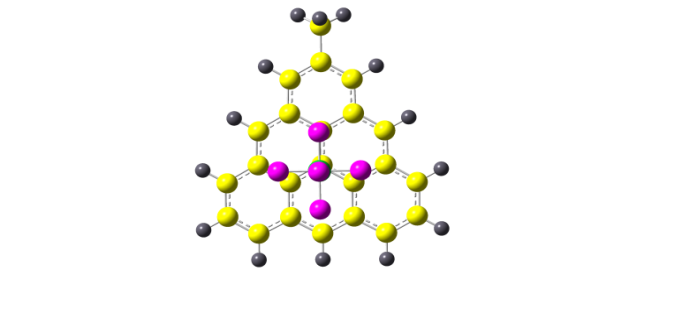

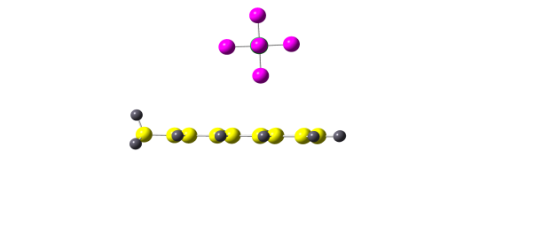


**Tri + CH_3_**


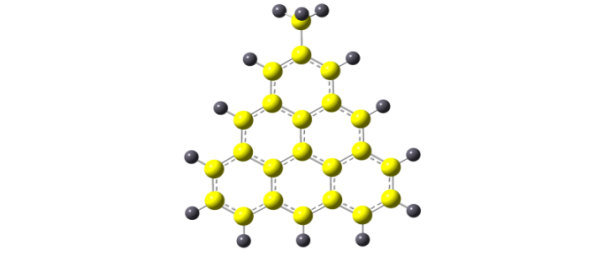


**Top View**

**Side View**

Figure S4: Optimized structures of SO_2_, SOF_2_, SO_2_F_2_, and SF_6_ with CH_3_ edge-functionalized triangulene with B3LYP functional.

**Tri +**

**COCH_3_+ SO_2_**

**Tri +**

**COCH_3_+ SOF_2_**

**Tri +**

**COCH_3_+ SO_2_F_2_**

**Tri +**

**COCH_3_+ SF_6_**


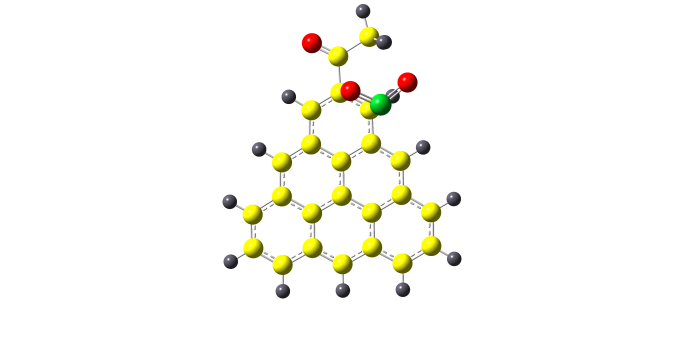

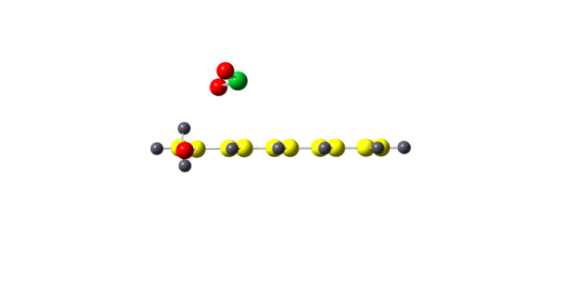

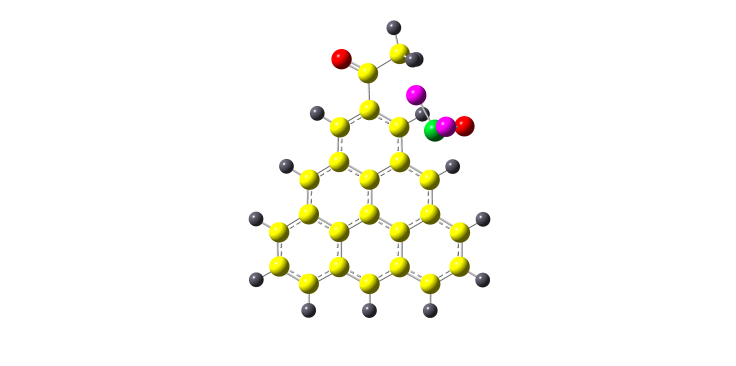

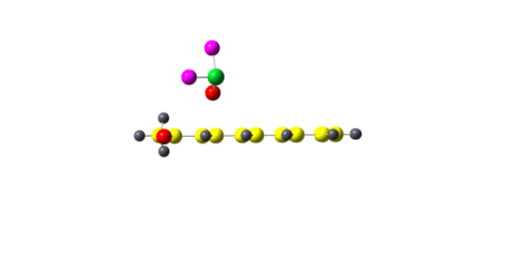

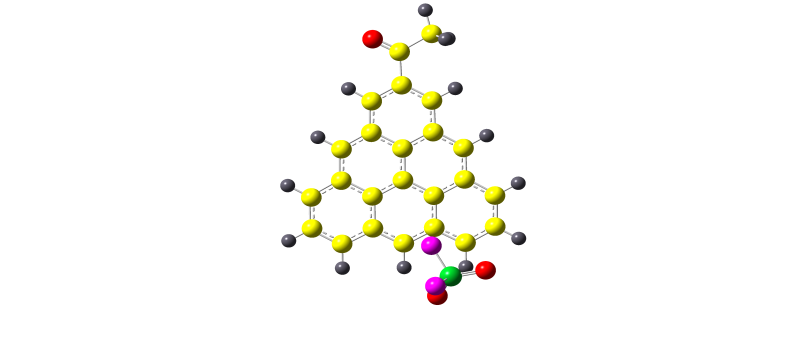

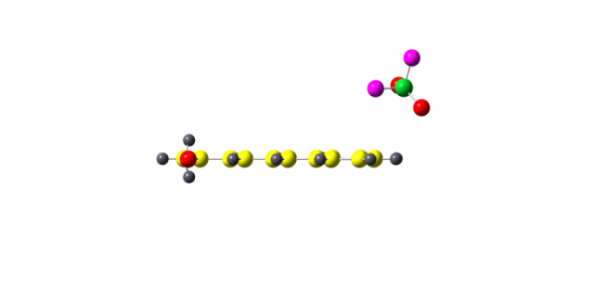

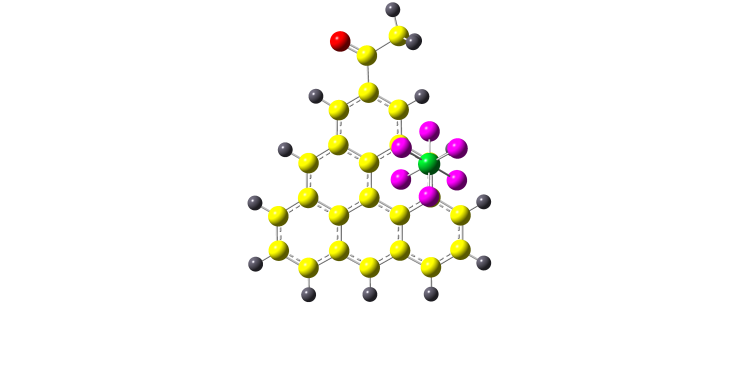

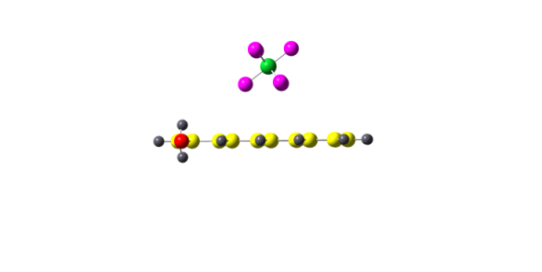


**Tri + COCH_3_**


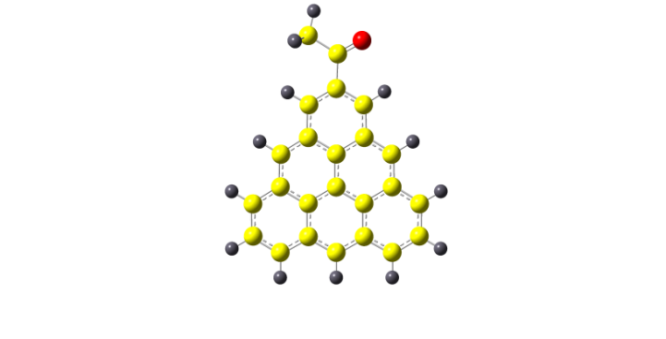


**Top View**

**Side View**

Figure S5: Optimized structures of SO_2_, SOF_2_, SO_2_F_2_, and SF_6_ with COCH_3_ edge-functionalized triangulene with B3LYP functional.

**Tri + NH_2_+ SO_2_**

**Tri+ NH_2_+ SOF_2_**

**Tri+ NH_2_+ SO_2_F_2_**

**Tri + NH_2_+ SF_6_**


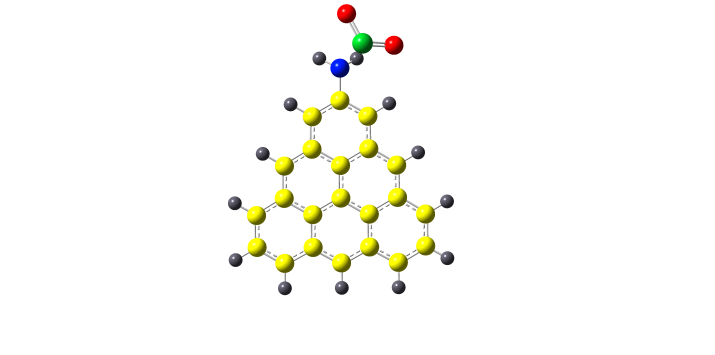

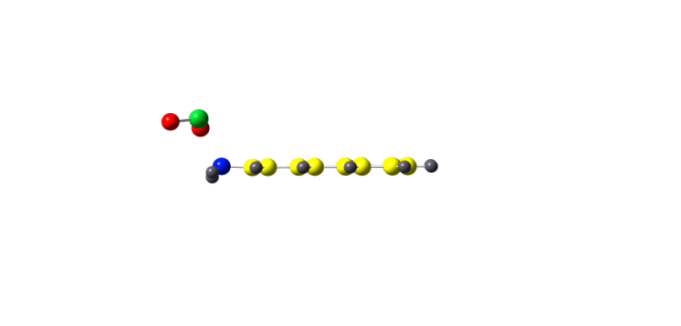

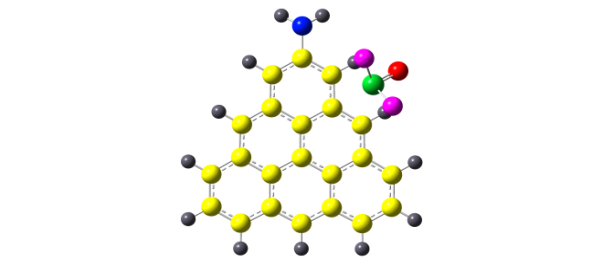

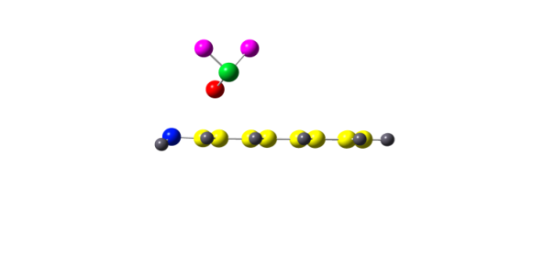

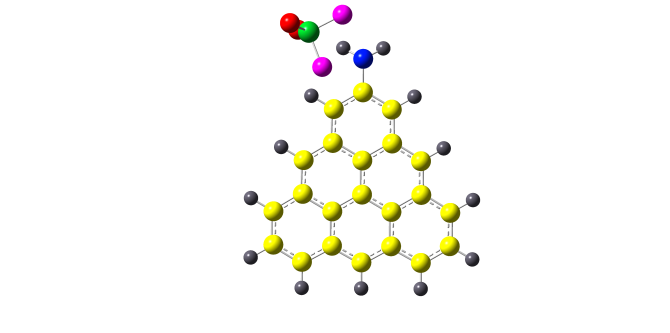

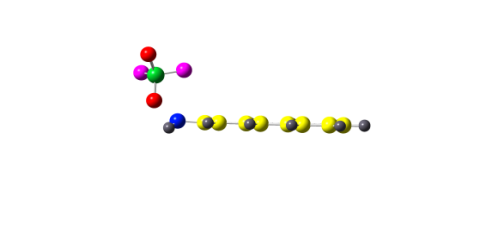

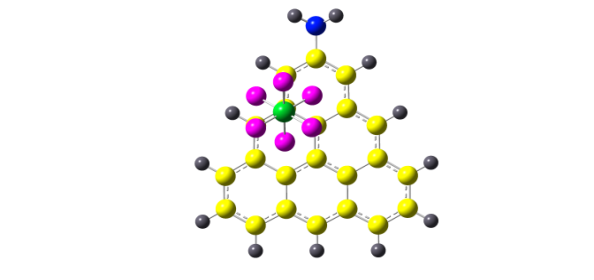

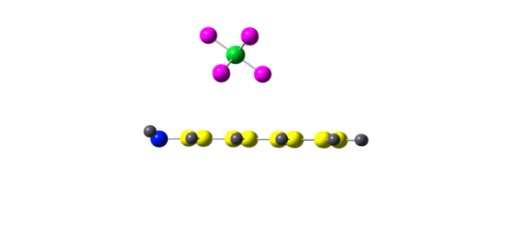


**Tri + NH_2_**


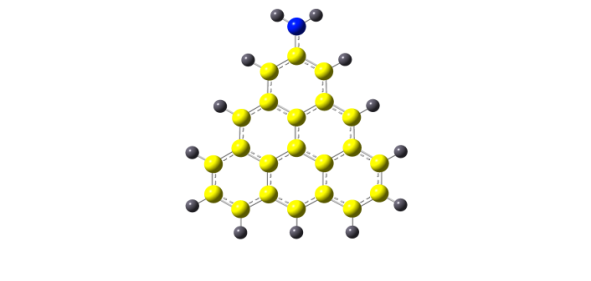


**Top View**

**Side View**

Figure S6: Optimized structures of SO_2_, SOF_2_, SO_2_F_2_, and SF_6_ with NH_2_ edge-functionalized triangulene with B3LYP functional.


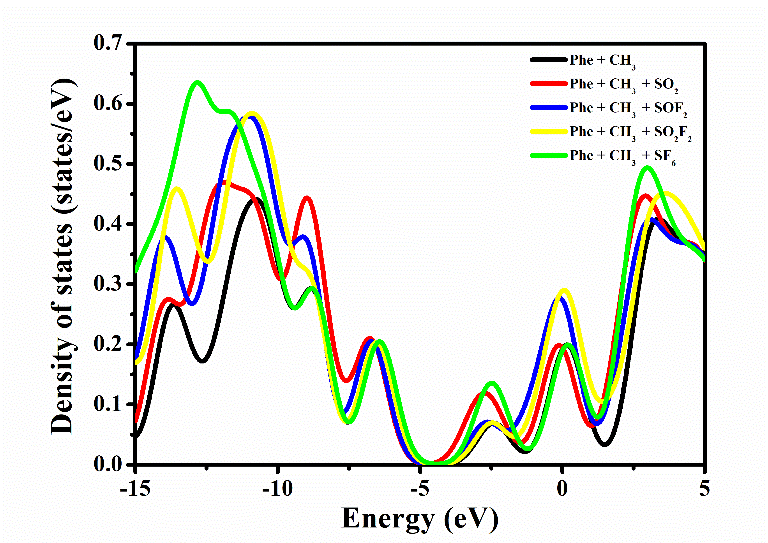

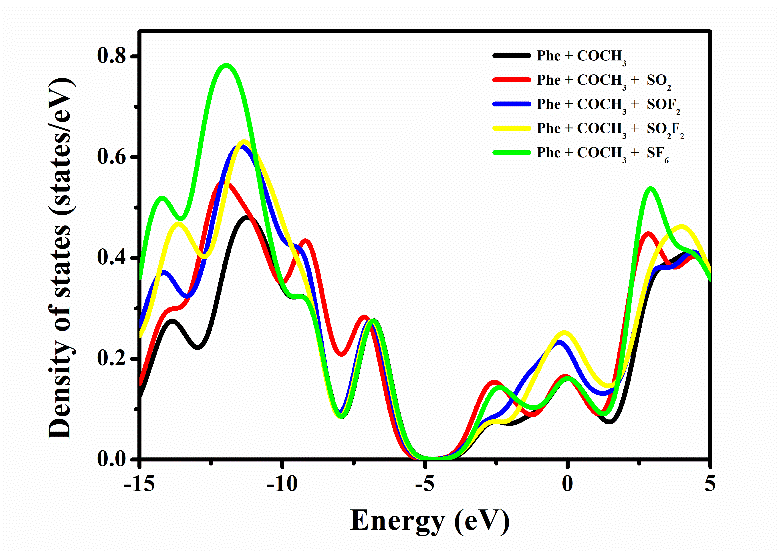

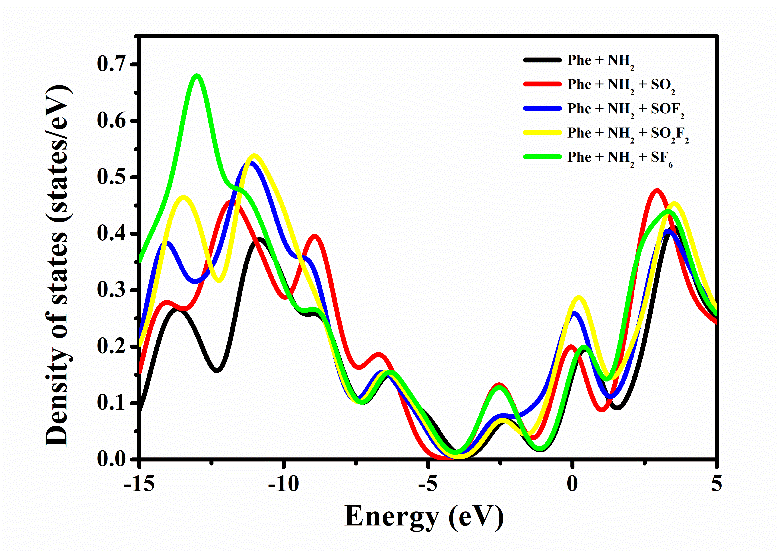

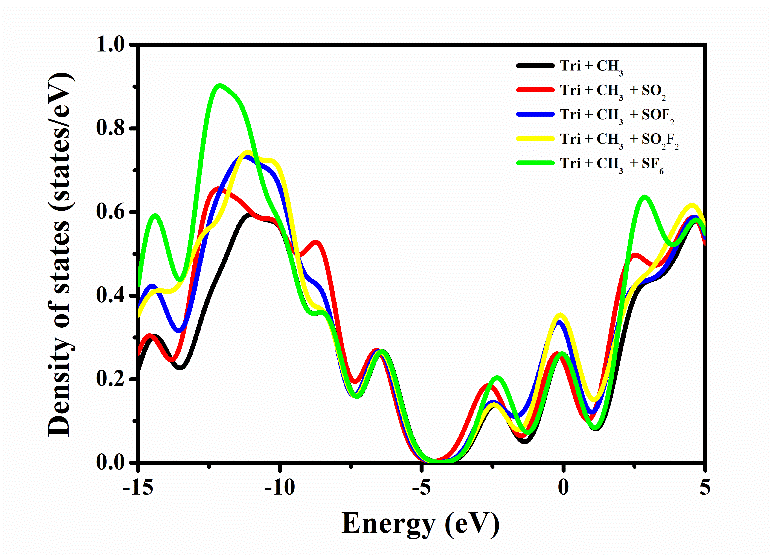

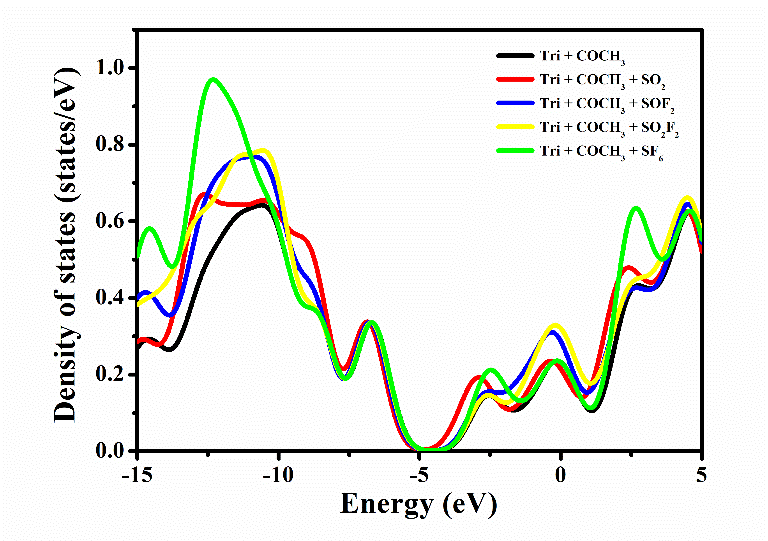

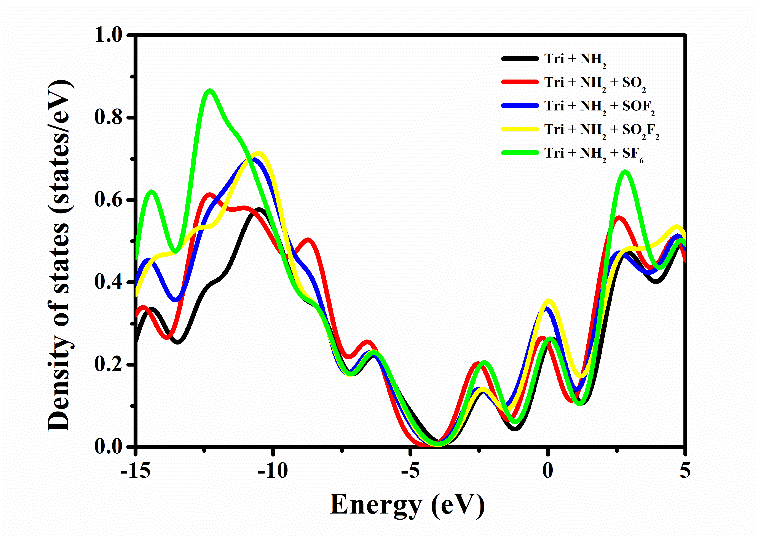


**(a)**

**(b)**

Figure S7: Density of states of adsorption of SO_2_, SOF_2_, SO_2_F_2_, and SF_6_ with (a) phenalenyl and (b) triangulene systems with B3LYP functional.


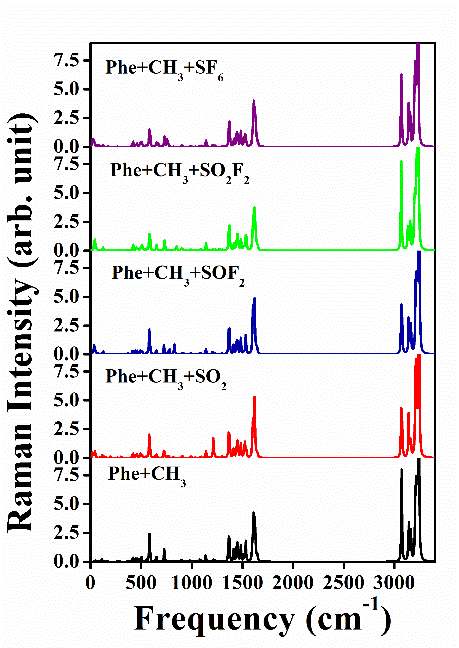

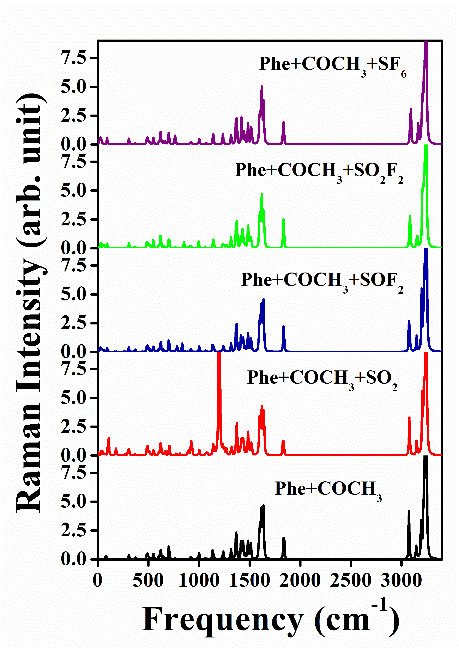

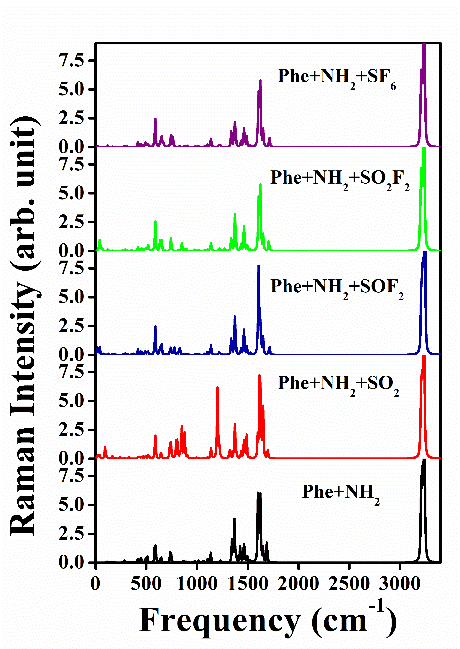

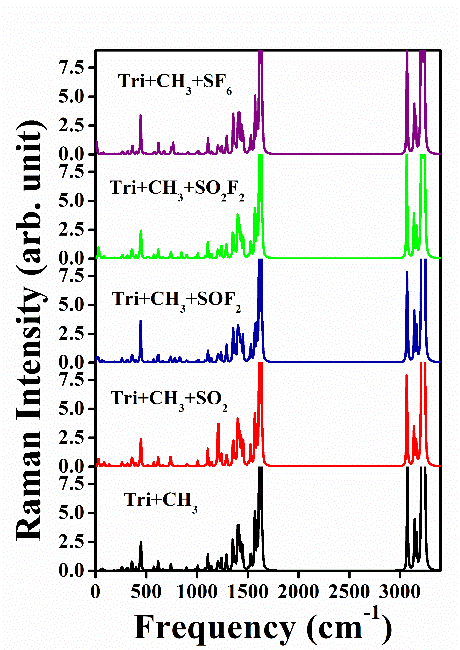

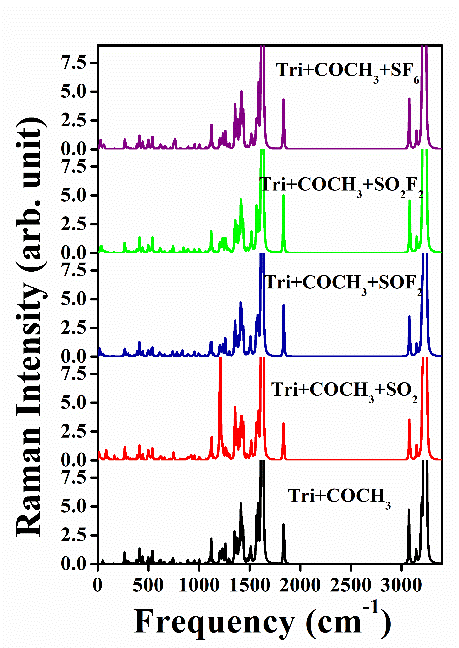

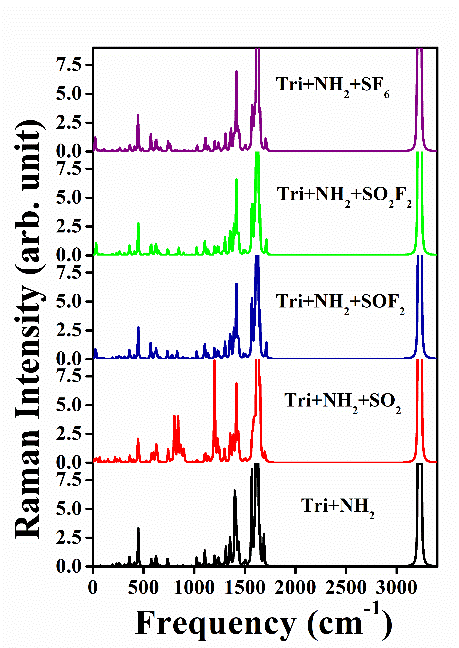

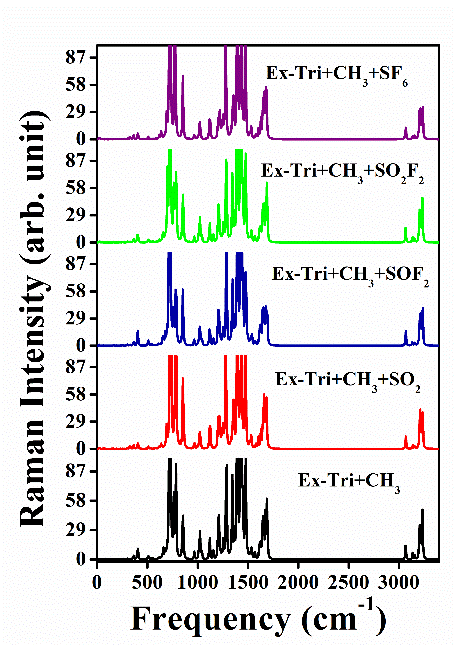

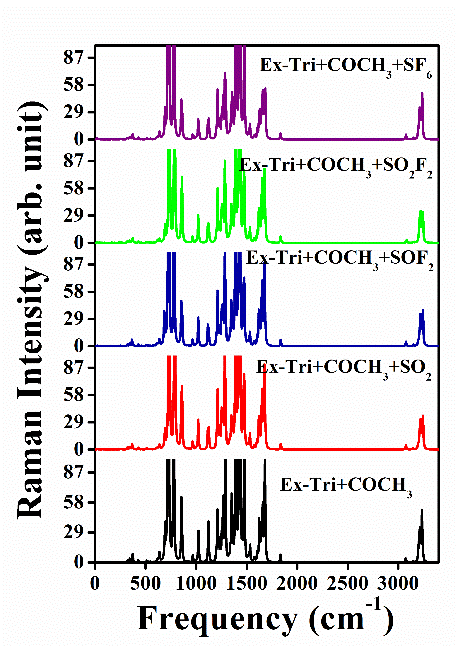

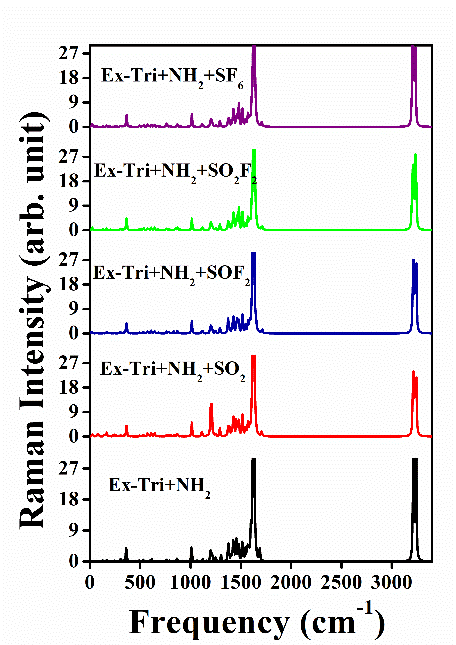


Figure S8: Raman plot of adsorption of SO_2_, SOF_2_, SO_2_F_2_, and SF_6_ with phenalenyl (upper row) and triangulene (middle row) and extended triangulene (lower row) systems with wB97XD functional.

**Basis set effect on adsorption energy:-**

We have also done the comparative study on the adsorption energy using higher basis set for all considered GQDs with 6-31G (D) basis set. We found, in our present study, the E_ad_ value with 6-31G (D) basis set is higher as compared to def2-TZVPP triple zeta basis set with single-point calculations. It is possible for the adsorption energy to be lower when using the def2-TZVPP triple zeta basis set as compared to the 6-31G (D) basis set, although the magnitude of the difference will depend on the individual systems being studied. The adsorption energy is a function of many factors, including the electronic structure of the adsorbate and substrate, as well as the adsorption geometry. It is also important to note that the adsorption energy is influenced by many other factors, such as the choice of functional and the accuracy of the geometry optimization. The E_ad ­­_values obtained using def2-TZVPP triple zeta basis set are tabulated in Table S1.

**Table S1: Adsorption Energy (E_ad_) of structure of SO_2_, SOF_2_, SO_2_F_2_, and SF_6_ adsorption on phenalenyl, triangulene and extended triangulene using def2-TZVPP basis set.**

| Structure | E_ad_ (eV) | Structure | E_ad_ (eV) | Structure | E_ad_ (eV) |
| --- | --- | --- | --- | --- | --- |
| Phe+CH_3_ | - | Tri+CH_3_ | - | Ex-Tri+CH_3_ | - |
| Phe+CH_3_+SO_2_ | -0.22 | Tri+CH_3_+SO_2_ | -0.23 | Ex-Tri+CH_3_+SO_2_ | -0.23 |
| Phe+CH_3_+SOF_2_ | -0.21 | Tri+CH_3_+SOF_2_ | -0.22 | Ex-Tri+CH_3_+SOF_2_ | -0.21 |
| Phe+CH_3_+SO_2_F_2_ | -0.15 | Tri+CH_3_+SO_2_F_2_ | -0.18 | Ex-Tri+CH_3_+SO_2_F_2_ | -0.18 |
| Phe+CH_3_+SF_6_ | -0.12 | Tri+CH_3_+SF_6_ | -0.11 | Ex-Tri+CH_3_+SF_6_ | -0.19 |
| Phe+COCH_3_ | - | Tri+COCH_3_ | - | Ex-Tri+COCH_3_ | - |
| Phe+COCH_3_+SO_2_ | -0.20 | Tri+COCH_3_+SO_2_ | -0.21 | Ex-Tri+COCH_3_+SO_2_ | -0.21 |
| Phe+COCH_3_+SOF_2_ | -0.19 | Tri+COCH_3_+SOF_2_ | -0.22 | Ex-Tri+COCH_3_+SOF_2_ | -0.21 |
| Phe+COCH_3_+SO_2_F_2_ | -0.15 | Tri+COCH_3_+SO_2_F_2_ | -0.19 | Ex-Tri+COCH_3_+SO_2_F_2_ | -0.18 |
| Phe+COCH_3_+SF_6_ | -0.13 | Tri+COCH_3_+SF_6_ | -0.17 | Ex-Tri+COCH_3_+SF_6_ | -0.19 |
| Phe+NH_2_ | - | Tri+NH_2_ | - | Ex-Tri+NH_2_ | - |
| Phe+NH_2_+SO_2_ | -0.33 | Tri+NH_2_+SO_2_ | -0.33 | Ex-Tri+NH_2_+SO_2_ | -0.27 |
| Phe+NH_2_+SOF_2_ | -0.25 | Tri+NH_2_+SOF_2_ | -0.26 | Ex-Tri+NH_2_+SOF_2_ | -0.27 |
| Phe+NH_2_+SO_2_F_2_ | -0.19 | Tri+NH_2_+SO_2_F_2_ | -0.22 | Ex-Tri+NH_2_+SO_2_F_2_ | -0.23 |
| Phe+NH_2_+SF_6_ | -0.13 | Tri+NH_2_+SF_6_ | -0.17 | Ex-Tri+NH_2_+SF_6_ | -0.23 |

1. Corresponding Author:

   Email addresses: [shukla@phy.iitb.ac.in](mailto:shukla@phy.iitb.ac.in) (A. Shukla), [oshivaishali@gmail.com](mailto:oshivaishali@gmail.com) (V. Roondhe), [rajeev.ahuja@physics.uu.se](mailto:rajeev.ahuja@physics.uu.se) (R. Ahuja) [↑](#footnote-ref-1)
